# Control of Bacterial Phenotype and Chromosomal Gene Expression by Single Plasmids of *Lactococcus lactis* IL594

**DOI:** 10.3390/ijms24129877

**Published:** 2023-06-08

**Authors:** Katarzyna Kosiorek, Anna Koryszewska-Bagińska, Marek Skoneczny, Tamara Aleksandrzak-Piekarczyk

**Affiliations:** 1 Institute of Biochemistry and Biophysics, Polish Academy of Sciences (IBB PAS), Pawińskiego 5a, 02-106 Warsaw, Poland; k.izdebska@ibb.waw.pl (K.K.); kicia@ibb.waw.pl (M.S.); 2 Department of Medical Biology, Medical University of Warsaw, Litewska 14/16, 00-575 Warsaw, Poland; akoryszewska@wum.edu.pl

**Keywords:** *Lactococcus lactis* IL594, *L. lactis* IL1403, plasmid, single-plasmid derivative, phenotypic and transcriptomic microarrays

## Abstract

Plasmid-free *Lactococcus lactis* IL1403 is one of the best-characterized representatives of lactic acid bacteria (LAB), intensively used in broad microbiology worldwide. Its parent strain, *L. lactis* IL594, contains seven plasmids (pIL1–pIL7) with resolved DNA sequences and an indicated role for overall plasmid load in enhancing host-adaptive potential. To determine how individual plasmids manipulate the expression of phenotypes and chromosomal genes, we conducted global comparative phenotypic analyses combined with transcriptomic studies in plasmid-free *L. lactis* IL1403, multiplasmid *L. lactis* IL594, and its single-plasmid derivatives. The presence of pIL2, pIL4, and pIL5 led to the most pronounced phenotypic differences in the metabolism of several carbon sources, including some β-glycosides and organic acids. The pIL5 plasmid also contributed to increased tolerance to some antimicrobial compounds and heavy metal ions, especially those in the toxic cation group. Comparative transcriptomics showed significant variation in the expression levels of up to 189 chromosomal genes due to the presence of single plasmids and 435 unique chromosomal genes that were resultant of the activity of all plasmids, which may suggest that the observed phenotypic changes are not only the result of a direct action of their own genes but also originate from indirect actions through crosstalk between plasmids and the chromosome. The data obtained here indicate that plasmid maintenance leads to the development of important mechanisms of global gene regulation that provide changes in the central metabolic pathways and adaptive properties of *L. lactis* and suggest the possibility of a similar phenomenon among other groups of bacteria.

## 1. Introduction

*Lactococcus lactis* is of great economic importance due to its wide use as the main constituent of dairy starter cultures, especially in the production of cheese and probiotic fermented milk [1]. In addition to extensive use in the food industry, lactococcal strains have also been used, among others, as bioreactors for the production of molecules of medical importance or vehicles for the delivery of DNA vaccines [2,3,4]. Extensive research has been conducted in the past on industrial features of *L. lactis* with particular emphasis on the metabolism of lactose, citrate, and casein and resistance to bacteriophages or heavy metals, all of which are often plasmid-related traits [5]. Because of the technological value of genes carried by plasmids, significant effort has been put into characterizing lactococcal plasmids, which are common components of the genome of *L. lactis* dairy isolates [6,7]. These mobile self-replicating extrachromosomal DNA molecules are not essential for the growth of the host, but they may affect its performance in the dairy industry. They can contribute to survival, adaptation to environmental changes, and even the conquest of new habitats, but at the same time, by replication of the plasmids or expression of genes encoded by plasmids, they may cause metabolic burden [8].

*L. lactis* subsp. *lactis* IL1403, a plasmid-free derivative of *L. lactis* IL594 strain, is one of the best-characterized Gram-positive microorganisms, widely used in biological research worldwide [9]. *L. lactis* IL594 was isolated from a cheese starter culture, indicating its important industrial use [10]. It harbors seven plasmids (pIL1 to pIL7) that contain genes relevant for technological processes and adaptation to specific environmental conditions. These genes are involved in lactose utilization (*lacR-lacABCDFEGX*), peptide degradation (*pepE* and *pepF*), oligopeptide uptake (*oppDFBCA*), citrate uptake (*citQRP*), bacteriophage resistance (*hsdS*, *hsdM*, *hsdR* genes of a type I restriction-modification system), and stress resistance (*cadAC*) [7].

Given the significance of lactococcal plasmids, in our previous study, we evaluated the impact of pIL1–pIL7 plasmids on overall chromosomal gene expression and global changes in host cell metabolic activity using high-throughput RNA and phenotypical microarrays (Biolog^®^). The presence of plasmids alters the expression of chromosomal genes, mainly with metabolic, defense, or regulatory functions. Differentially expressed genes are involved in carbohydrate and global energy metabolism, the transport and metabolism of amino acids, nucleotides, and inorganic ions, and defense mechanisms. The presence of the gene pool from pIL1–pIL7 plasmids, through the slightly increased tolerance to a range of antimicrobial compounds, improves the overall fitness and metabolic capacity of the host [11]. While the plasmid control of chromosomally encoded genes is not unusual, only a few reports on detailed plasmid–chromosome crosstalk analyses can be found, and data on this phenomenon in lactic fermentation bacteria in particular are lacking.

This prompted us to undertake further experiments using derivatives of the multiplasmid *L. lactis* strain IL594 to identify changes caused by a specific plasmid. Our hypothesis was that adaptive and industrially important metabolic traits of plasmid-bearing strains may be determined directly by the presence of plasmid genes or/and indirectly by affecting the genome fingerprint via plasmid–chromosome crosstalk. The results presented here indicate the role of individual plasmids by identifying chromosomal gene activity and phenotypical changes as a result of their influence, significantly enhancing the current knowledge of bacterial plasmidome activity.

## 2. Results

### 2.1. Presence of Plasmids Increases Ability to Utilize Carbon Compounds

**Figure 1:**
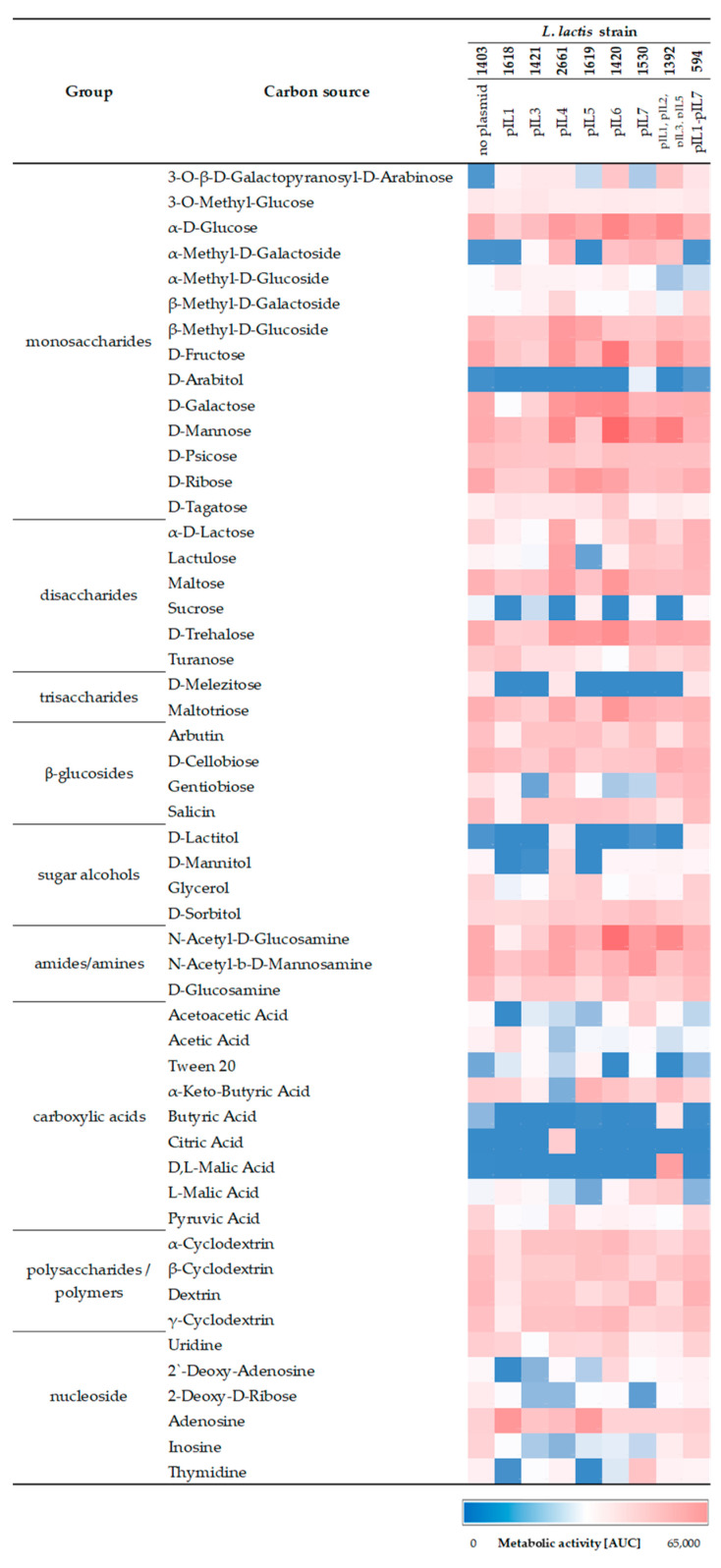
Phenotypic MicroArray for carbon source utilization by *L. lactis* strains carrying plasmids or plasmid-free *L. lactis* IL1403. Colors from blue to red correspond to area under the curve (AUC) values of metabolic activity. Only those carbon sources are shown on which metabolic activity of at least one strain was detected, and the colorimetric reaction kinetics plot had an AUC > 15,000 in Omnilog arbitrary units (OAU).

The utilization of carbon sources in plasmid-free *L. lactis* IL1403, single-plasmid *L. lactis* (IL1618 (pIL1 plasmid), IL1421 (pIL3 plasmid), IL2661 (pIL4 plasmid), IL1619 (pIL5 plasmid), IL1420 (pIL6 plasmid), IL1530 (pIL7 plasmid)), and multiplasmid *L. lactis* (IL1392 (pIL1–pIL3 and pIL5 plasmids), IL594 (pIL1–pIL7 plasmids)) was analyzed by two assays—API 50CH and Phenotype MicroArrays. Both *L. lactis* IL1403 and single-plasmid derivatives of IL594 were obtained using a protoplast-induced curing method as described previously by Chopin, A. et al. [12]. In the present study, the stability of plasmid content during experimental procedures was confirmed by the polymerase chain reaction (RT-PCR) with the use of primers specifically exclusive for a particular plasmid. All strains were able to efficiently ferment 14 of the 49 mono- and disaccharides present in API 50CH (D-galactose, D-glucose, D-fructose, D-maltose, D-mannose, N-acetylglucosamine, D-ribose, D-trehalose, arbutin, cellobiose, and salicin). Compared to plasmid-free *L. lactis* IL1403, the metabolism of the other three glycosides (esculin, gentiobiose, and lactose) was increased in *L. lactis* IL2661 and IL594. Between 190 different carbon sources from Phenotype MicroArrays (amino acids, amides, amines, carbohydrates, β-glucosides, esters, fatty acids, sugar alcohols, and carboxylic acids), in 45 of them, at least one lactococcal strain showed notable metabolic activity (area under the curve (AUC) above 1.5 × 10^4^ in Omnilog Arbitrary Units (OAU)) (Figure 1). In favor of plasmid-containing strains, the most noticeable phenotypic differences were noted during the metabolism of C-sources, including 3-0-β-D-galactopyranosyl-D-arabinose, malic acid, butyric acid, α-methyl-D-galactoside, D-lactitol, adenosine, gentiobiose, D-lactose, and lactulose with particular emphasis on the first five substrates, which were not used at all by the plasmid-free strain. For the other carbon sources, the differences in metabolism between *L. lactis* IL1403 and plasmid-positive strains were less significant or absent (Figure 1). The strongest metabolic differences were observed in strains IL1392 and IL2661—the presence of four plasmids in the former enabled the metabolism of organic acids (citric, malic, butyric) and enhanced the utilization of α,β-methyl-D-galactosides and disaccharides (gentiobiose lactose, lactulose), while in the second one with pIL4, the metabolism of disaccharides (lactose, lactulose, gentiobiose) and the increased metabolic activity of disaccharide derivative (lactitol) were observed.

### 2.2. Presence of pIL5 Enhances L. lactis Tolerance to Antimicrobial Compounds

**Figure 2:**
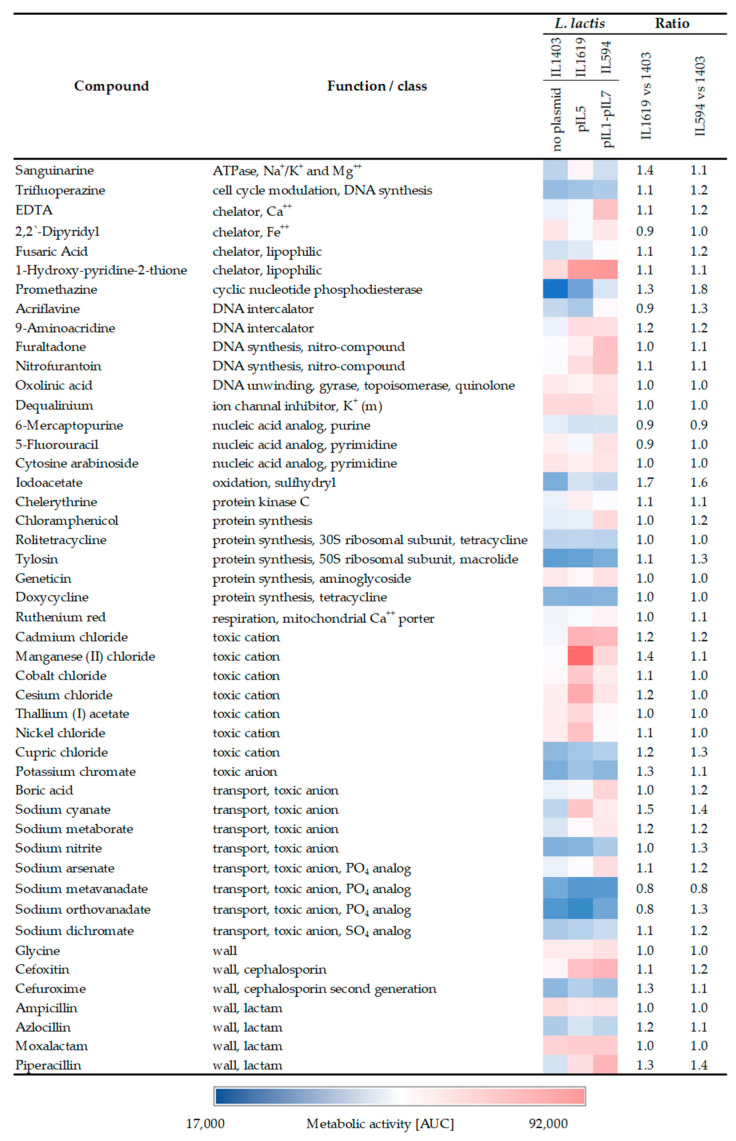
Changes in the tolerance of various compounds between *L. lactis* IL594, IL1619, and plasmid-free IL1403. The colors from blue to red show the average values of the areas under the curve (AUC) of metabolic activity recorded at four different concentrations of the compounds. The recorded metabolic activity ranged from 17,000 OAU to 92,000 OAU (Omnilog Arbitrary Units), with the lowest values indicating no metabolic activity of the strain. Ratios were calculated as the quotient of the metabolic activity of plasmid-positive and plasmid-free *L. lactis* strains.

Among the strains tested and compared to plasmid-free *L. lactis* IL1403, IL1619 and IL594 showed the most pronounced changes in tolerance to several chemical compounds (Figure 2). The most apparent effect on the resistance of plasmid-containing strains was identified in the presence of toxic ions such as cadmium chloride, manganese (II) chloride, sodium cyanate, or sodium arsenate, in the presence of which there was an approximately 20–50% increase in their metabolic activity (Figure 2). In the vast majority of cases, the presence of plasmids had no significant effect on the antibiotic tolerance of analyzed *L. lactis* strains. The most pronounced, but only at most 40%, increase in the activity of plasmid strains was observed for the most part in the presence of some representatives of the β-lactam group (cefoxitin, cefuroxime, piperacillin) (Figure 2). Metabolic activity coefficients in plasmid-containing strains were also generally slightly elevated for some other compounds, including chelators (EDTA, 1-Hydroxy-Pyridine-2-thione), ATPases (sanguarine), and nitro compounds (furaltadone, nitrofurantoin), but the observed differences were subtle and did not exceed 40%. In many cases, the enhancement of tolerance to chemical compounds was similar between the multiplasmid strain and IL1619 containing only pIL5, which may suggest that of the pool of all plasmids of strain IL594, it is pIL5 that determines the occurrence of this phenomenon.

### 2.3. The Presence of Single Plasmids Changes the Expression of Chromosomal Genes

In the comparative transcriptomic analysis between plasmid harboring *L. lactis* strains and plasmid-free IL1403, a range of 42 to 189 changes in chromosomal gene expression caused by single-plasmid presence was detected (Table 1), representing from 2% to 9% of all annotated chromosomal genes in the *L. lactis* IL1403 genome. The total number of identified unique genes reached 435. The most prominent changes were observed in the IL1392 strain harboring four plasmids (pIL1, pIL2, pIL3, and pIL5) with 148 upregulated and 41 down-regulated chromosomal genes. Conversely, the presence of pIL7 plasmid in the IL1530 strain led to the least variability in chromosomal gene expression with only 25 up- and 17 downregulated genes. In all analyses of single-plasmid *L. lactis* strains, the presence of plasmids more often activated than inhibited chromosomal gene expression (Table 1).

**Figure 3:**
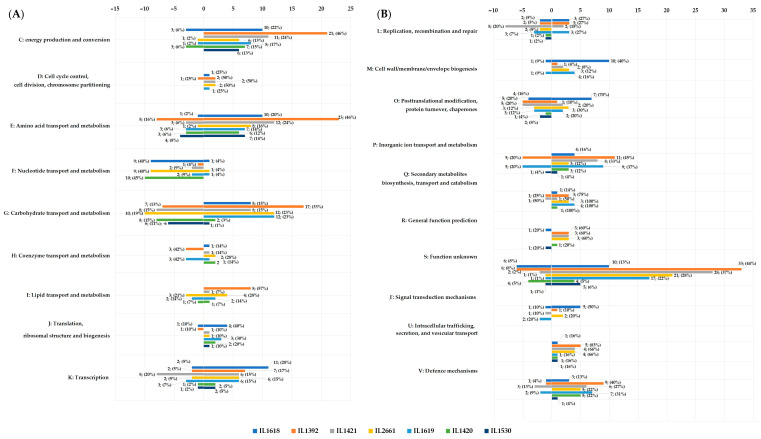
Changes identified in chromosomal gene expression profiles in *L. lactis* strains with single plasmids within the COG category. Negative numbers at the top of the graph refer to the number of chromosomal genes repressed in the presence of plasmids in a given strain; positive numbers refer to the number of identified genes with increased expression in the presence of plasmid genes in a given strain. Percentages refer to the proportion of genes identified in the analyzed strains to the total number of unique genes with altered expression profiles from the distinguished COG category. (**A**) Genes from C-K COG functional categories; (**B**) genes from L–V COG functional categories.

Chromosomal genes differentially expressed in the presence of pIL1–pIL7 plasmids were grouped based on their biological role according to functional categories of COGs (clusters of orthologous groups) (Supplementary Table S1). The presence of extrachromosomal replicons led to prominent changes in the expression of chromosomal genes, mainly with metabolic, defense, or regulatory functions. More than 49% of the identified changes (212 differentially expressed unique genes) corresponded to genes of metabolic functions from seven COGs, including energy production and conversion (C), the transport and metabolism of carbohydrates (G), amino acids I, nucleotides (F), inorganic transport (P), lipids (I), and coenzymes (H) (Figure 3). Genes encoding proteins of unknown function (COG cat. S) were strongly represented, with 143 unique genes transcribed exclusively in the presence of plasmids (Supplementary Table S1).

Among genes with metabolic functions, a pool of 49 chromosomal genes with differential expression was assigned to the amino acid transport and metabolism (E) category (11% of all identified changes) (Figure 3, Supplementary Table S1), where the dominance of expression activation was observed in all analyzed strains (76% of transcriptomic changes). The strongest activation of expression of genes involved in oligopeptide transport (*opp* operon) (Log2Ratio 6.9–7.9) was observed in the presence of pIL4 plasmid (strain IL2661), while the most numerous changes were recorded for *L. lactis* IL1392, with the increased transcription of 23 genes, including dehydrogenase (*asd*), dehydratase (*ilvD*), peptidase involved in casein degradation (*pepF*), and a set of ABC transporters for mono-, di-, and tripeptides (i.e., *yddA*, *ysaBCD*, *yrfBD*). In the context of substrate transport, the upregulation of several genes (*ydgC, yshA*) (Log2Ratio 2.1–3.4) encoding secondary transport systems (uniporters and antiporters) that engaged in amino acid uptake was also observed in strain IL1392 (Supplementary Table S1).

The presence of four plasmids in strain IL1392 also led to a considerable variation in the expression profile of genes involved in energy production and conversion (the COG cat. C) and carbohydrate transport and metabolism (COG cat. “G”). Within these, the significant activation (Log2Ratio 2.1–4.6) of genes involved in citrate (*citBCD*), malate (*maa*, *mleS*), gluconate, and glutamate (*gltPQS*) metabolism was observed. Furthermore, the expression profile of genes involved in redox reactions was also altered and included those upregulated, such as several oxidoreductases (*ybiE*, *yddB*, *ymgK*, *yqcA*, *yugB*), acyl-dehydrogenase (*pdhB*), and those repressed encoding NADH/NADPH oxidases and reductases, such as *yiaD*. In the context of sugar transport and catabolism, the plasmids also activated (up to three-fold) the expression of genes participating in the phosphotransferase system (PTS)-driven uptake of fructose (*fruA*) and xylose/xyloside (*ywiH*, *xylT*, *xynTB*) (Supplementary Table S1). In contrast, the presence of plasmids caused the inhibition (Log2Ratio 1.9–3.9) of the expression of the entire Leloir pathway operon, encoding components of the complete galactose and lactose utilization system (*galPMKTE*-*thgA*-*lacZ*).

The plasmids altered the transcriptional profiles of 43 unique genes also from the COG categories associated with nucleotide (F), coenzyme (H), and lipid (I) transport and metabolism (Figure 3). The most abundant effects occurred in *L. lactis* IL2661 and IL1420, with the down-regulation (up to Log2Ratio 1.5–2.5) of F category genes, such as transferases (*pydB*, *pyrBE*), decarboxylase (*pyrF*), and the *xpt* gene involved in guanine metabolism. In the H category, the most noticeable decrease (Log2Ratio 2–3) was in the expression of folic acid synthase (*pabAB*) and thiamine synthase (*apbE*). In the group of genes involved in lipid transport and metabolism, *L. lactis* IL1392 and IL2661 had increased expression levels of genes encoding proteins involved in fatty acid degradation and unsaturated fatty acid synthesis, such as acetyl-CoA carboxylase (*accBCD*), ACP S-malonyltransferase (*fabD*), enoyl-ACP reductase (*fabI*), (3R)-hydroxymyristoyl-ACP dehydratase (*fabZ*), and long-chain acyl-CoA synthetase (*fadD*). On the other hand, probably to counterbalance this activation effect, the same long-chain acyl-CoA synthetase FadD appeared to be strongly (Log2Ratio almost 6) repressed in strain IL1619.

Among genes in the COG categories of inorganic ion transport and metabolism (P) and defense mechanism (V), the most notable changes in chromosomal gene expression profiles occurred in *L. lactis* IL1619 and IL1392, both carrying pIL5. Both strains had a similar pattern of identified changes in the transcriptome, with a strong predominance of expression activation. In the presence of pIL5, an increase of expression of genes mainly involved in the transport of mono- and bivalent cation such as potassium (*yjdJ*), cadmium (*cadA*), magnesium (*pacL*, *ypbB*), or heavy metals (*cbiO*, *ypbC*, *ytgB*, *yvfA*) was observed (Figure 3, Supplementary Table S1). The expression of multidrug efflux pumps (*yhcA*, *ypbC*, *yvhA*, *ywiG*, *yxbD*) was also markedly upregulated, as were components of drug transport systems, including the general drug antiporter (*lmrP*) and the gene encoding kanamycin kinase (*ymdC*), but the changes were subtle and did not exceed a Log2Ratio of 1.9 shift in transcript levels. In contrast, the expression of the *mtsABC* cluster encoding components of the manganese import pathway and ammonium transport system (*amtB*) were negatively regulated.

All *L. lactis* strains analyzed showed changes in the expression levels of genes involved in transcriptional regulation (COG category K). The expression of transcriptional regulators was clearly manipulated, with a predominance of activation in the presence of multiple plasmids, but this was especially evident in the presence of pIL1 or pIL3 carried by *L. lactis* IL1618 and IL1421, respectively. Distinct changes in the expression of transcriptional regulators were also seen in IL1392, which is probably the result of the joint action of pIL1 and pIL3, which, among others, occur together in this strain. Many of the plasmid-controlled transcription regulators do not yet have assigned functions but can be assigned to various families (TetR/AcrR, ArsR, DeoR, NadR, RpiR, Crp, MarR, LytR, Xre). Genes with elevated levels of transcription encode, among others, proteins with putative regulatory function (e.g., *ynaAB*, *rliABDE*, *yjaBJ*), fructose uptake repressor (*fruR*), ferric uptake regulator (*fur*), NAD biosynthesis regulator (*nadR*), or phosphate transport system regulator (*phoU*). In contrast, transcription regulators belonging to the TetR/AcrR, MarR, LytR, or Xre family were markedly downregulated, including regulators of fatty acid biosynthesis (*rmaA*, *rmaG*), hyaluronan biosynthesis (*rmaJ*), and galactose utilization (*glaR*/*yugA*) or those involved in heme homeostasis (*ygfC*) (Supplementary Table S1).

## 3. Discussion

Knowledge of the role of individual plasmids and/or their genes in crosstalk with the chromosome and subsequent changes in the expression of the bacterial phenotype is still poor. Here, in an approach of high-throughput phenotypic and transcriptomic analyses, we assigned a function to individual plasmids from the multiplasmid *L. lactis* IL594 regarding the modulation of host chromosome gene expression and phenotype. As part of these analyses, we identified a sizable set of changes in chromosomal gene expression levels, accounting for 20% of all genes in the chromosome of *L. lactis* under study. While some phenotypic changes may be encoded by plasmid genetic determinants per se, we postulated here that some are due to plasmid–chromosome crosstalk. A flagship example might be the increased transcription of the operon encoding the ABC transporter dedicated to oligopeptide uptake observed in the presence of certain plasmids. The entire *oppDFBCA* operon is located in pIL4 [7], and in strain IL2661, we observed an unusually strong increase in transcript levels, which is most likely due to the gene expression of this system from the pIL4 plasmid. However, a copy of the *oppDFBCA* operon was also located in the chromosome, and in IL1421 and IL1392 strains containing the pIL3 plasmid, the activation of the operon was evident, suggesting a pIL4-independent effect of pIL3 on bacterial protein metabolism by affecting the host chromosome.

Strains with plasmids showed a greater capacity for carbohydrate fermentation, resulting in the increased or even enabled use of several of them. The most obvious phenotypic differences within the analyzed plasmid and plasmid-free strains were observed for IL1392 (harboring pIL1, pIL2, pIL3, and pIL5 plasmids), IL2661 (harboring pIL4 plasmid), and IL1420 (harboring pIL6 plasmid). Increased use of monosaccharides such as glucose, fructose, and mannose was observed in these three strains, and since the plasmids residing in them do not carry genes involved in the metabolism of any of these sugars, this suggested that the effect of their increased utilization has a basis stemming from the plasmid–chromosome crosstalk. Indeed, the presence of pIL1 and pIL5 plasmids enhanced the transcription of the *fruRCA* operon, encoding a complete lactococcal fructose metabolism system consisting of the DeoR family regulator, 1-phosphofructokinase, and the IIABC component of fructose-specific PTS (PTS^fru^) [13]. In *L. lactis* IL1403, glucose and mannose were transported by another sytem, the mannose-specific PTS (PTS^man^), and it was previously shown that, in this strain, this is the only system specific for mannose and potentially glucose, as its inactivation leads to the full abolition of the ability of IL1403 to grow on mannose [14], and the alternative system for glucose transport used in other lactococci (PTS^cel^ encoded by *ptcABC*) [15] is inactive in IL1403 most likely due to the lack of expression of the PtcC permase (our unpublished study). However, the results of the transcriptomic microarray analysis here did not show an enhancement of the expression of the *ptnABCD* genes encoding PTS^man^ in the presence of any of the plasmids, which may suggest that another system with affinity for mannose and glucose may be regulated by the plasmids and involved in the transport of these two monosaccharides.

In a previous study, we indicated the role of the overall plasmid load in strain IL594 in enhancing the metabolism of more complex carbon compounds, such as certain β-glycosides [11]. Here, we specified that plasmids residing in strains IL2661 and/or IL1392 are mainly involved in this process, as their presence led to the enhancement of their hosts of the use of lactose and its derivatives as well as β-glucosides, such as esculin and gentiobiose. While the efficient metabolism of lactose, lacticol, and lactulose in strain IL2661 is certainly due to the activity of the pIL4-encoded lactose-specific PTS system (PTS^lac^) [7], the genetic determinants that condition the β-glucoside metabolism appeared to be absent in plasmids residing in strains IL2661 and IL1392. However, a glance at chromosomal gene expression data indicates an upregulation of the expression level of the *ypcA* gene encoding a hypothetical P-β-glucosidase in these two strains. Confirmation of the role of the novel P-β-glucosidase YpcA in the hydrolysis of esculin and gentiobiose in *L. lactis* and the specific plasmidic genetic determinants leading to its elevated expression levels requires further study.

Among other chromosomal genes involved in sugar metabolism, notable changes induced by the presence of single plasmids were reported here in the expression of those relevant to galactose, xyloside, or xylose metabolism. Galactose metabolism in *L. lactis* IL403 has already been deciphered to involve solely transport by GalP permease (LacS) and subsequent transformation in the Leloir pathway, with the entire operon subject to activation by the RpiR-family transcriptional regulator GlaR (YugA) [16]. Here we showed that in the presence of pIL3 or pIL7 plasmids, the *glaR* gene was repressed, resulting in the reduced transcription of the entire Leloir operon, as observed for its *galPMKTE-thgA-lacZ* genes. However, the repression of the Leloir operon did not become obvious at the phenome level, as no definite reduction in galactose utilization was observed in plasmid-carrying strains, suggesting a rather loose control of GlaR activator expression by plasmidic genes. Similarly, changes in the expression of chromosomal *xylAT* genes did not become apparent at the phenomic level; despite their activation caused by the presence of plasmids pIL3, pIL4 (*xylT*), or pIL5 (*xylA*), the strain remained xylose-negative. However, this phenomenon finds its simple explanation in a mutation in the *xylT* gene encoding the D-xylose proton symporter, leading to the shortening of the C-terminus of the protein and, consequently, its inactivation and the inability of *L. lactis* IL1403 to grow on xylose, despite the presence of a complete operon for the metabolism of this sugar [17]. Thus, it can be concluded that carrying pIL3-, pIL4-, or pIL5-like mobile elements in Xyl^+^ bacteria will result in the improved efficiency of utilization of this carbon source and to the advantage of their hosts in the plant environment.

The effect of crosstalk between the plasmid and the chromosome was also apparent in the regulation of genes involved in dihydroxyacetone (DHA) use. The *dhaLM* genes encoding dihydroxyacetone kinase subunits were overexpressed in IL1619, suggesting a possible role for plasmid pIL5 in the metabolic regulation of DHA conversion to glyceraldehyde-3-phosphate and entry into the glycolysis pathway [18]. The mechanism of *dhaLM* gene upregulation may be directly related to the enhanced expression of the *dhaK* transcription activator gene also observed in the presence of the pIL5 plasmid in multiplasmid strains of *L. lactis*. These observations present an interesting possibility of the plasmid- and chromosome-associated intensification of DHA bioproduction in *L. lactis*, especially in the context of the increasing use of DHA in drugs and cosmetics [19].

Increased tolerance to antimicrobial compounds and metal ions represents an important mechanism of LAB environmental adaptation. Here, we showed that the presence of plasmids in *L. lactis* led to subtle differences in the tolerance of theirs hosts to several chemical compounds, with most changes observed in strain IL1619 with pIL5, confirming our previous suspicion of its importance in enhancing cell resistance in the multiplasmid *L. lactis* IL594 [11]. These findings imply that pIL5 may play a role in increasing cell defense by the action of its own resistance genes and/or by regulating the expression of the chromosomal ones. Indeed, the increased tolerance to heavy metals can be directly linked to the presence of plasmidic genes located in pIL5, such as those encoding components of the Cd^2+^/Ni^2+^ ion transport system (*cad* operon). This operon contains regulatory *cadX* and resistance *cadD* genes, encoding the metalloid efflux system with specificity to Cd^2+^, Co^2+^, and Zn^2+^ [20]. The phenotype of increased *L. lactis* resistance to heavy metal ions through efflux transporters, mostly chromosomally encoded, have been previously reported [21]. However, adaptation to toxic ions may also result from the plasmid-derived influence on chromosomal gene expression, as we observed altered the expression of many chromosomal genes from the COG category of inorganic ion transport. Among them, the transcription of *cadA*, *cbiO*, *pacL*, and *yuhE*, encoding components of mono- and divalent cation efflux systems (Cs^+^, Co^2+^, Zn^2+^), were most strongly activated. Interestingly, the phenotypic effect of enhanced tolerance to cesium, cadmium, and cobalt ions was observed in IL1619 and IL594, supporting our previous speculation of the important role of plasmid pIL5 in enhancing the cellular tolerance of *L. lactis* [11]. Here, we also confirmed the previously described phenomenon of plasmid-induced repression of all three components of the *mtsABC* operon encoding the manganese ABC transport system in *L. lactis* IL594 [10]. Manganese is an essential cofactor required for appropriate enzymatic functions of stress response and cell growth [22], and some genes encoding putative transporters located in pIL5 can also enhance manganese ion uptake (e.g., putative Mn^2+^ and Fe^2+^ transporter of the NRAMP family encoded by the *orf16* gene). However, a potential toxic effect through the accumulation of manganese in the cytoplasm has also been described, highlighting the need for the accurate regulation of its homeostasis [23]. This study indicated that pIL5 reduces the activity of the *mtsABC* system, and thus, the cell is protected from excess manganese. As support for this hypothesis, we identified a pIL5-dependent increase in tolerance to manganese ions in both IL1619 and IL594 strains compared to plasmid-free IL1403, indicating a benefit for pIL5-containing cells in dealing with possible excess of this metal.

The pIL5 plasmid may also have a role in increasing the tolerance of susceptible *L. lactis* cells to some antibiotics and other antimicrobial compounds, but only small changes of at most 40% have been observed in its presence that do not lead to antibiotic resistance, where there is a multiple increase in the level of resistance compared to the wild-type strain [24,25,26]. One mechanism of resistance is the presence of highly conserved multidrug resistance (MDR) efflux pumps that remove antibiotics outside the bacterial cell, and at least 20 genes encoding antibiotic-related transporters have been identified in the genome of susceptible *L. lactis* IL1403 [9]. Here, we showed that the presence of plasmids can induce changes in the expression levels of some of them, including *ywiG* and *ypbC* encoding putative multidrug resistance transporters of the ABC family, the expression of which was elevated in the presence of plasmids pIL3 and pIL4. The genes *ymdC* encoding an aminoglycoside resistance protein, *yvhA*, and *lmrP*-encoding MDR efflux pumps were also upregulated in the presence of plasmid pIL5. Of these genes, only *lmrP* functionality in *L. lactis* has so far been confirmed to confer resistance to clinically important antibiotics [27,28]. It cannot be ruled out that plasmids may influence other mechanisms of increased antibiotic tolerance, such as cell envelope remodeling. In IL1618 and IL1619 strains, there was the activation of some genes of the *dlt* operon involved in the incorporation of d-alanine in teichoic acids (TA), which is one of the mechanisms of resistance to various positively charged antimicrobials in Gram-positive bacteria [29,30,31]. This observation seems interesting in view of the activation, also observed in IL1618 and IL1619, of *ysaACB* genes encoding an ABC transporter that in *L. lactis* is one of the components of the peptide-sensing and detoxification (PSD) module [32,33]. Previously, it was pointed out that the important function of activation of this module in conditioning resistance to peptide antimicrobial compounds is realized through the induction of *dlt* operon expression [33], and here, we showed the role of pIL1 and pIL5 in supporting this resistance mechanism.

The role of plasmids cannot be overestimated due to the fact that they increase host adaptive functions, with some of their properties resulting not only from the direct activity of the genes they carry but also from indirect actions through modulation of the expression of relevant chromosomal genes. In the case of *L. lactis* IL594 and its single-plasmid derivatives, the presence of individual plasmids led to an increase in host adaptive functions, with a subtle trend at both genotypic and phenotypic levels. Further studies should focus on the identification and characterization of specific plasmid genes that are responsible for effects on chromosomal gene expression observed here and consequently the corresponding phenotypic changes.

## 4. Materials and Methods

### 4.1. Bacterial Strains and Growth Conditions

*L. lactis* strains used in this study are shown in Table 2. Bacteria were grown aerobically at 30 °C in M17 (Oxoid Ltd., Basingstoke, Hampshire, UK) supplemented with 1% (*w*/*v*) cellobiose (M17-Cel). Solidified media contained 1.5% agar (Sigma-Aldrich, Saint Louis, MO, USA).

### 4.2. Metabolic Activity and Resistance Assays

Metabolic profiles were evaluated globally using the Phenotype MicroArrays (PM) system (Biolog Inc., Hayward, CA, USA), according to the manufacturer’s instructions. *L. lactis* strains were cultured on M17-Cel solid medium, and the grown colonies were suspended in IF0a inoculating fluid until the solution reached the transmittance of 81%. Growth additives and tetrazolium redox dye D (Biolog, USA) were added corresponding to standard protocols recommended by Biolog for *Streptococcus* spp. A total of 100 μL of aliquot was added to each well in PM panels and incubated in an OmniLog incubator-reader (30 °C; 96 h). Colorimetric data were collected approximately every 15 min and analyzed using OmniLog PM software version 2.3.01 (Biolog, USA). 

### 4.3. Oligonucleotide Microarrays

Prior to RNA isolation, confirmation of the presence of plasmids in *L. lactis* IL594 and its single-plasmid derivatives was carried out using polymerase chain reaction (PCR) with primers specific to selected unique plasmid genes (Supplementary Table S3). The total RNA was isolated from 10 mL *of L. lactis* cultures grown in M17 medium supplemented with cellobiose from mid-exponential growth phase (OD_600_ 0.6–0.7) using GeneMATRIX Universal RNA Purification Kit (EURx, Gdańsk, Poland), according to the manufacturer’s protocol. For cDNA MicroArrays transcriptomics experiments, 100 ng of total RNA was used as the template for providing Cy3 or Cy5 fluorescently labeled cDNA by the use of the Agilent Quick Amp Two-Color Labeling Kit (Agilent Technologies, Santa Clara, CA, USA) according to the manufacturer’s protocol. The comparative microarray hybridizations of plasmid-harboring and IL1403 strains transcriptomes were performed in two biological replicates. Hybridizations were performed with two technical replicates with dye-swap. Custom Gene Expression 8x15 K Microarray slides (Design ID: 084868) containing 2441 60-mer oligonucleotide probes representing all known *L. lactis* IL594 genes were used (Agilent Technologies, USA). The fluorescence images obtained were further scanned with the Axon GenePix 4000B (Molecular Devices, San Jose, CA, USA) microarray scanner. Feature extraction was achieved with GenePix Pro 6.1. The raw data were Lowess-normalized, averaged, and statistically analyzed using Acuity 4.0 software (Molecular Devices). A LogRatio value expressing the over- or underrepresentation of the transcript in compared samples was calculated for each analyzed gene. A gene was identified as differentially expressed between compared *L. lactis* strains if the level of its transcript differed by|Log2Ratio| ≥ 1.5 with the statistical significance, *p* < 0.05 (Supplementary Table S1). In order to be able to refer to the off-threshold data, an additional transcriptomic data set with relaxed thresholds of Log2Ratio ≥ 1.2) and statistical significance *p* < 0.05 was also developed (Supplementary Table S2). The subsequent lists of differentially expressed genes (DEGs) were subjected to bioinformatic analysis. ORF annotations of *L. lactis* were obtained from the BASys Omix 4.0 software [34]. Differentially expressed genes were grouped based on putative functions of the encoded proteins into COG categories using the WebMGA RPS-blast tool (Weizong-Lab) (http://weizhong-lab.ucsd.edu/webMGA/server/cog/, accessed on 15 January 2023). Due to the different results between biological repeats, both in this work and in our previous studies [11], in the current paper, we did not present or include differently regulated genes from the COG X category (Mobilome: prophages, transposons) in the comparisons. The complete transcriptome analysis data were deposited in the Gene Expression Omnibus (https://www.ncbi.nlm.nih.gov/geo/, accessed on 12 April 2023) under accession no: GSE229414.

## Figures and Tables

**Table 1:** Numbers of differentially expressed genes in plasmid harboring *L. lactis* strains.

*L. lactis* Strain	IL1618	IL1421	IL2661	IL1619	IL1420	IL1530	IL1392	IL594
Plasmid (s)	pIL1	pIL3	pIL4	pIL5	pIL6	pIL7	pIL1, pIL2, pIL3, pIL5	pIL1–pIL7
Upregulated genes	90	98	89	88	37	25	148	21
Downregulated genes	30	33	30	28	31	17	41	23
Total genes affected	120	131	119	116	68	42	189	44
% of chromosomal genes	5%	6%	5%	5%	3%	2%	9%	2%

**Table 2:** Strains used in this study.

*L. lactis* Strain	Genotypic Characteristics	Source
IL594	wild-type strain carrying seven plasmids: pIL1–pIL7	INRA
IL1618	carrying pIL1 plasmid	INRA
IL1392	carrying four plasmids: pIL1, pIL2, pIL3, pIL5	INRA
IL1421	carrying pIL3 plasmid	INRA
IL2661	carrying pIL4 plasmid	INRA
IL1619	carrying pIL5 plasmid	INRA
IL1420	carrying pIL6 plasmid	INRA
IL1530	carrying pIL7 plasmid	INRA
IL1403	plasmid-free strain, derivative of IL594	INRA

INRA—strains collection of National Institute of Agricultural Research, France.

## Data Availability

The complete transcriptome analysis data were deposited in the Gene Expression Omni-bus (https://www.ncbi.nlm.nih.gov/geo/) under accession no: GSE229414).

## Supplementary Materials

The supporting information can be downloaded at: https://www.mdpi.com/article/10.3390/ijms24129877/s1.

## References

[1] Wels M., Siezen R., Van Hijum S., Kelly W.J., Bachmann H. (2019). Comparative genome analysis of *Lactococcus lactis* indicates niche adaptation and resolves genotype/phenotype disparity. Front. Microbiol..

[2] Tavares L.M., de Jesus L.C.L., da Silva T.F., Barroso F.A.L., Batista V.L., Coelho-Rocha N.D., Azevedo V., Drumond M.M., Mancha-Agresti P. (2020). Novel strategies for efficient production and delivery of live biotherapeutics and biotechnological uses of *Lactococcus lactis*: The Lactic Acid Bacterium model. Front. Bioeng. Biotechnol..

[3] De Castro C.P., Drumond M.M., Batista V.L., Nunes A., Mancha-Agresti P., Azevedo V.A.C. (2018). Vector development timeline for mucosal vaccination and treatment of disease using *Lactococcus lactis* and design approaches of next generation food grade plasmids. Front. Microbiol..

[4] Batista V.L., da Silva T.F., de Jesus L.C.L., Tapia-Costa A.P., Drumond M.M., Azevedo V., Mancha-Agresti P. (2021). Lactic Acid Bacteria as delivery vehicle for therapeutics applications. Methods Mol. Biol..

[5] Ainsworth S., Stockdale S., Bottacini F., Mahony J., van Sinderen D. (2014). The *Lactococcus lactis* plasmidome: Much learnt, yet still lots to discover. FEMS Microbiol. Rev..

[6] Van Mastrigt O., Di Stefano E., Hartono S., Abee T., Smid E.J. (2018). Large plasmidome of dairy *Lactococcus lactis* subsp. *lactis* biovar diacetylactis FM03P encodes technological functions and appears highly unstable. BMC Genom..

[7] Górecki R.K., Koryszewska-Bagińska A., Gołębiewski M., Żylińska J., Grynberg M., Bardowski J.K. (2011). Adaptative potential of the *Lactococcus lactis* IL594 strain encoded in its 7 plasmids. PLoS ONE.

[8] Kobayashi M., Nomura M., Fujita Y., Okamoto T., Ohmomo S. (2002). Influence of lactococcal plasmid on the specific growth rate of host cells. Lett. Appl. Microbiol..

[9] Bolotin A., Wincker P., Mauger S., Jaillon O., Malarme K., Weissenbach J., Ehrlich S.D., Sorokin A. (2001). The complete genome sequence of the lactic acid bacterium *Lactococcus lactis* ssp. *lactis* IL1403. Genome Res..

[10] Johansen E. (2003). Challenges when transferring technology from *Lactococcus* laboratory strains to industrial strains. Genet. Mol. Res..

[11] Kosiorek K., Koryszewska-Bagińska A., Skoneczny M., Stasiak-Różańska L., Aleksandrzak-Piekarczyk T. (2023). The presence of plasmids in *Lactococcus lactis* IL594 determines changes in the host phenotype and expression of chromosomal genes. Int. J. Mol. Sci..

[12] Chopin A., Chopin M.C., Moillo-Batt A., Langella P. (1984). Two plasmid-determined restriction and modification systems in *Streptococcus lactis*. Plasmid.

[13] Barrière C., Veiga-da-Cunha M., Pons N., Guédon E., van Hijum S.A.F.T., Kok J., Kuipers O.P., Ehrlich D.S., Renault P. (2005). Fructose Utilization in *Lactococcus lactis* as a model for low-GC Gram-positive bacteria: Its regulator, signal, and DNA-binding Site. J. Bacteriol..

[14] Tymoszewska A., Diep D.B., Wirtek P., Aleksandrzak-Piekarczyk T. (2017). The non-lantibiotic bacteriocin Garvicin Q targets Man-PTS in a broad spectrum of sensitive bacterial genera. Sci. Rep..

[15] Castro R., Neves A.R., Fonseca L.L., Pool W.A., Kok J., Kuipers O.P., Santos H. (2009). Characterization of the individual glucose uptake systems of *Lactococcus lactis*: Mannose-PTS, cellobiose-PTS and the novel GlcU permease. Mol. Microbiol..

[16] Aleksandrzak-Piekarczyk T., Szatraj K., Kosiorek K. (2019). GlaR (YugA)-a novel RpiR-family transcription activator of the Leloir pathway of galactose utilization in *Lactococcus lactis* IL1403. Microbiologyopen.

[17] Siezen R.J., Starrenburg M.J.C., Boekhorst J., Renckens B., Molenaar D., van Hylckama Vlieg J.E.T. (2008). Genome-scale genotype-phenotype matching of two *Lactococcus lactis* isolates from plants identifies mechanisms of adaptation to the plant niche. Appl. Environ. Microbiol..

[18] Christen S., Srinivas A., Bähler P., Zeller A., Pridmore R., Bieniossek C., Baumann U., Erni B. (2006). Regulation of Dha operon of *Lactococcus lactis* A deviation from the rule followed by the TetR family of transcription regulators. J. Biol. Chem..

[19] Stasiak-Różańska L., Kupiec M. (2018). Industrial applications of wild and genetically-modified strains of acetic acid bacteria. Postępy Mikrobiol.-Adv. Microbiol..

[20] Crupper S.S., Worrell V., Stewart G.C., Iandolo J.J. (1999). Cloning and expression of *cadD*, a new cadmium resistance gene of *Staphylococcus aureus*. J. Bacteriol..

[21] Monachese M., Burton J.P., Reid G. (2012). Bioremediation and tolerance of humans to heavy metals through microbial processes: A potential role for probiotics?. Appl. Environ. Microbiol..

[22] Pajarillo E.A.B., Lee E., Kang D.-K. (2021). Trace metals and animal health: Interplay of the gut microbiota with iron, manganese, zinc, and copper. Anim. Nutr..

[23] Nelson N. (1999). Metal ion transporters and homeostasis. EMBO J..

[24] Nawaz M., Wang J., Zhou A., Ma C., Wu X., Moore J.E., Millar B.C., Xu J. (2011). Characterization and transfer of antibiotic resistance in lactic acid bacteria from fermented food products. Curr. Microbiol..

[25] Alvarez Y., Ponce-Alquicira E. (2018). Antibiotic resistance in Lactic Acid Bacteria. Antimicrobial Resistance-a Global Threat.

[26] Gad G.F.M., Abdel-Hamid A.M., Farag Z.S.H. (2014). Antibiotic resistance in lactic acid bacteria isolated from some pharmaceutical and dairy products. Braz. J. Microbiol..

[27] Lubelski J., Mazurkiewicz P., van Merkerk R., Konings W.N., Driessen A.J.M. (2004). *ydaG* and *ydbA* of *Lactococcus lactis* encode a heterodimeric ATP-binding cassette-type multidrug transporter. J. Biol. Chem..

[28] Poelarends G.J., Mazurkiewicz P., Konings W.N. (2002). Multidrug transporters and antibiotic resistance in *Lactococcus lactis*. Biochim. Biophys. Acta.

[29] Kramer N.E., van Hijum S.A.F.T., Knol J., Kok J., Kuipers O.P. (2006). Transcriptome analysis reveals mechanisms by which *Lactococcus lactis* acquires nisin resistance. Antimicrob. Agents Chemother..

[30] Giaouris E., Briandet R., Meyrand M., Courtin P., Chapot-Chartier M.-P. (2008). Variations in the degree of alanylation of teichoic acids in *Lactococcus lactis* alter resistance to cationic antimicrobials but have no effect on bacterial surface hydrophobicity and charge. Appl. Environ. Microbiol..

[31] McBride S.M., Sonenshein A.L. (2011). The *dlt* operon confers resistance to cationic antimicrobial peptides in *Clostridium difficile*. Microbiology.

[32] Campelo A.B., López-González M.J., Escobedo S., Janzen T., Neves A.R., Rodríguez A., Martínez B. (2020). Mutations selected after exposure to bacteriocin Lcn972 activate a bce-like bacitracin resistance module in *Lactococcus lactis*. Front. Microbiol..

[33] Tymoszewska A., Ovchinnikov K.V., Diep D.B., Słodownik M., Maron E., Martínez B., Aleksandrzak-Piekarczyk T. (2021). *Lactococcus lactis* resistance to Aureocin A53- and enterocin L50-like bacteriocins and membrane-targeting peptide antibiotics relies on the YsaCB-KinG-LlrG four-component system. Antimicrob. Agents Chemother..

[34] Van Domselaar G.H., Stothard P., Shrivastava S., Cruz J.A., Guo A., Dong X., Lu P., Szafron D., Greiner R., Wishart D.S. (2005). BASys: A web server for automated bacterial genome annotation. Nucleic Acids Res..

