# Supplementary material for: Control of Bacterial Phenotype and Chromosomal Gene Expression by Single Plasmids of Lactococcus lactis IL594
Source: Int J Mol Sci. 2023 Jun 8;24(12):9877. doi: 10.3390/ijms24129877 (PMC10298230; doi:10.3390/ijms24129877)
Supplement: Supplementary file 1 [file ijms-24-09877-s001.zip › ijms-2379329-supplementary.pdf]

Control of bacterial phenotype and chromosomal gene expression by single plasmids of *Lactococcus lactis* IL594

Katarzyna Kosiorek[1], Anna Koryszewska-Bagińska[2], Marek Skoneczny[1], and Tamara Aleksandrzak-Piekarczyk[1,*]

[1] Institute of Biochemistry and Biophysics, Polish Academy of Sciences (IBB PAS), Pawińskiego 5a, 02-106 Warsaw, Poland; K.K.: k.izdebska@ibb.waw.pl; M.S.: kicia@ibb.waw.pl; T.A.P: tamara@ibb.waw.pl
[2] Department of Medical Biology, Medical University of Warsaw, Litewska 14/16, 00-575 Warsaw, Poland; A.K.B.: akoryszewska@wum.edu.pl
* Correspondence: tamara@ibb.waw.pl; +48 592 12 13

Supplementary Materials

Table S1. Differentially expressed genes within plasmid-containing *L. lactis* strains and plasmid-free derivative IL1403. A gene was identified as differentially expressed between compared *L. lactis* strains if the level of its transcript differed by|Log2Ratio|≥1.5 with the statistical significance, $p<0.05$. Differentially expressed genes were grouped based on putative functions of the encoded proteins into COG categories. In comparative transcriptomic analysis, a range of 42 to 189 changes in chromosomal genes expression caused by single-plasmid presence were detected, representing from 2% to 9% of all annotated chromosomal genes in the *L. lactis* IL1403 genome. The total number of identified unique genes reached 435.

Gene	NCBI GeneID	COG	Gene upregulation or downregulation *L. lactis* strain								Protein function
			**IL1618**	**IL1421**	**IL2661**	**IL1619**	**IL1420**	**IL1530**	**IL1392**	**IL594**	
Plasmid / plasmids			**pIL1**	**pIL3**	**pIL4**	**pIL5**	**pIL6**	**pIL7**	**pIL1 pIL2 pIL3 pIL5**	**pIL1-pIL7**	
**Energy production and conversion**											
adhE	WP_129881180.1	C			3.2		1.7	2.1	2.2		bifunctional acetaldehyde-CoA
agl	CP009472.1	C							4.8		alpha-glucosidase
citB	AY268077.1	C		1.8		2.0		2.3	2.5		acetate-SH-citrate lyase
citC	AY268077.1	C							2.3		acetate-SH-citrate lyase

citD	AY268077.1	C							3.0		citrate lyase subunit gamma
gltA	NC_002662.1	C				2.1		1.6			citrate synthase
gltP	NC_009004.1	C		1.8		1.5	3.0		2.1		glutamate ABC transporter permease
gltQ	NC_002662.1	C					2.9	1.8	1.9		glutamate ABC transporter ATP-binding protein
gltS	NC_017486.1	C		1.9		2.3	3.2		3.0		glutamate or arginine ABC transporter substrate binding protein
ipd	NC_017486.1	C				1.6					indole-3-pyruvate decarboxylase
maa	NC_020450.1	C							4.6		maltose O-acetyltransferase
mleP	NC_020450.1	C		3.0					3.2		malate transporter
mleS	AAK04998.1	C		3.0					2.9		malate dehydrogenase
msmK	NC_017486.1	C								-2.6	multiple sugar ABC transporter ATP-binding protein
pdhB	NC_020450.1	C							2.1		PDH E1 component subunit beta
pydA	NC_002662.1	C			1.8						dihydroorotate dehydrogenase 1A
pyrZ	AAK05445.1	C			-2.0		-2.4				dihydroorotate dehydrogenase electron transfer subunit
trxA	NZ_CP059048.1	C	2.6								thioredoxin
trxB1	NZ_CP059048.1	C	1.8								thioredoxin reductase
tyrS	WP_058204081.1	C	-1.7			-2.5					tyrosyl-tRNA synthetase
ybiE	GFO78088.1	C	1.9						3.3		oxidoreductase
yddB	AAK04431.1	C							2.0		oxidoreductase
yfdE	NP_389061.1	C	-1.8								hypothetical protein similar to myo-inositol monophosphatases
yfhC	AAK04676.1	C	2.6		1.5	3.7					hypothetical protein
ygaJ	NP_388758.2	C	2.0								putative peptidase
yiaD	AAK04894.1	C	3.4		1.9				2.1		NADH-flavin reductase
yjcA	YP_026228.1	C		1.7							ABC transporter ATP-binding protein
yjgC	NP_389098.1	C								-1.5	amino acid ABC transporter substrate binding protein
ylbA	NP_459521.1	C	1.6								ABC transporter ATP-binding protein
ymgG	NZ_CM001513.1	C		1.7							hypothetical protein

ymgH	NZ_CM001513.1	C		1.6				1.5		hypothetical protein
ymgK	YP_009518766.1	C	1.5						2.4	oxidoreductase
yobA	NP_416355.1	C	1.9			1.7				hypothetical protein
yogA	NP_389725.1	C					-1.7			hypothetical protein
yphC	NP_417040.2	C					-3.9			oxidoreductase
yqcA	NP_417270.1	C							1.7	oxidoreductase
yrjA	NZ_CP059048.1	C	-1.5							hypothetical protein
yrjB	AAK05831.1	C		1.6	1.5		2.0	1.9		oxidoreductase
yrjC	NZ_CP059048.1	C					1.7			iron-binding oxidase subunit
yugB	NP_268143.1	C							2.3	oxidoreductase
ywdA	NP_268313.1	C							1.8	hypothetical protein
ywdC	NP_268315.1	C		1.7					1.7	hypothetical protein
ywiD	AAK06296.1	C	2.9							hypothetical protein
ywiH	AAK06302.1	C		2.8	2.2		1.6		3.3	ABC transporter permease
ywjF	NP_268372.1	C				1.6				3-hydroxyisobutyrate dehydrogenase
**Cell cycle control, cell division, chromosome partitioning**										
ezrA	NC_022369.1	D	2.4			1.8				septation ring formation regulator EzrA
scpA	NP_267433.1	D							-1.6	segregation and condensation protein A
yihA	NP_267005.1	D		1.8	1.8				1.8	hypothetical protein
yihB	NP_267006.1	D		1.7	1.6				1.5	hypothetical protein
**Amino acid transport and metabolism**										
arcA	WP_004254504.1	E	1.8				3.0	2.8	2.5	arginine deiminase
arcB	AJ001103.1	E		1.7			2.8	2.2	2.3	ornithine carbamoyltransferase
arcC1	AAK06131.1	E					2.7			carbamate kinase
arcC2	AAK06131.1	E	2.6	1.7			2.6			carbamate kinase
arcD1	U81991.1	E		1.8			3.2	1.9	2.1	arginine/ornitine antiporter
argF	NC_022369.1	E		1.7					1.7	ornithine carbamoyltransferase

aroH	NC_000913.3	E				-2.0					phospho-2-dehydro-3-deoxyheptonate aldolase
asd	NC_000913.3	E							2.3		aspartate-semialdehyde dehydrogenase
bmpA	AE005176.1	E							-2.2		basic membrane protein A
ceo	U23376.1	E					1.7				N5-carboxyethyl-ornithine synthase
cysD	AAK04171.1	E							-2.2		O-acetylhomoserine sulfhydrylase
cysK	QOK50869.1	E		-2.5			-2.6	-1.6	-2.7		cysteine synthase serine family
cysM	NC_049805.1	E		-3.0			-2.9	-1.7	-2.4		cysteine synthase
glnB	AAK05689.1	E						2.1			nitrogen regulatory protein P-II
glnQ	SPS12229.1	E								-1.9	glutamine ABC transporter ATP-binding protein
hisA	NC_022369.1	E						1.5			1-(5-phosphoribosyl)-5-[(5-phosphoribosylamino)methylideneamino] imidazole-4-carboxamide isomerase
ilvD	SPS12837.1	E							2.2		dihydroxy-acid dehydratase
leuC	NC_022369.1	E						1.5			isopropylmalate isomerase large subunit dehydratase component
lysP	NC_020450.1	E		2.0					2.3		lysine specific permease
mapA	NC_002662.1	E	2.8						5.2		maltose phosphorylase
metB1	NC_017486.1	E	-1.5								cystathionine gamma-synthase
metB2	NC_020450.1	E		-2.8	-1.6		-2.4	-1.6	-3.2		cystathionine gamma-synthase
oppA	QQF01346.1	E		1.8					2.4		oligopeptide ABC transporter substrate binding protein
oppB	QQF01347.1	E		3.2	7.6				3.8	6.4	oligopeptide ABC transporter permease
oppC	QQF01348.1	E			7.9	2.0			2.0	6.3	oligopeptide ABC transporter permease
oppD	QQF01345.1	E			5.9					5.0	oligopeptide ABC transporter ATP binding protein
oppF	QQE99754.1	E		1.5	6.9				1.8	5.7	oligopeptide ABC transporter ATP binding protein
optS	NC_002662.1	E		1.8		2.3		1.5	2.2		oligopeptide ABC transporter substrate binding protein
pepDB	NC_002662.1	E	1.7								dipeptidase
pepF	CAA83534.1	E							2.0	1.6	oligoendopeptidase F
potA	NC_000913.3	E							-1.8		spermidine/putrescine ABC transporter ATP-binding protein

poxL	NC_009004.1	E	2.8	1.7	1.8	2.7			2.3		pyruvate oxidase
serB	NP_266761.1	E			1.6						phosphoserine phosphatase
ybeC	NP_266300.1	E	1.9							1.6	hypothetical protein
yceJ	NP_266406.1	E				1.6					hypothetical protein
ycjI	WP_226319220.1	E							-1.9		hypothetical protein
yddA	NP_266488.1	E		2.4	2.6				3.0		transporter transport and binding protein
ydgC	NP_266512.1	E							1.8		amino acid permease
yibG	AAK04907.1	E				-1.6					amino acid permease transport and binding protein
ylcC	NP_267269.1	E	2.0								hypothetical protein
ylcF	NP_267272.1	E		2.0					1.6		hypothetical protein
yneH	NP_267469.2	E			2.5						hypothetical protein
yrfB	NP_267851.1	E							1.8		NADH-dependent oxidoreductase
yrfD	AAK05797.1	E							1.8		amino acid antiporter
ysaB	NP_267905.1	E	3.7			3.0			2.2		ABC transporter permease and substrate binding protein
ysaC	NP_267906.1	E	3.0			2.8			2.1		ABC transporter ATP-binding protein
ysaD	NP_267907.1	E	2.8			2.9			1.6		hypothetical protein
yshA	AAK05907.1	E				-2.1		-1.6			glutamate antiporter
ysjA	NP_267979.1	E							-2.2	-1.6	amino acid permease transport and binding protein
**Nucleotide transport and metabolism**											
apt	AE005176.1	F	-2.5								adenine phosphoribosyltransferase
carA	NC_022369.1	F			-2.3		-1.7				carbamoyl phosphate synthase small subunit
carB	AJ000109.1	F			-1.9		-2.3				carbamoyl phosphate synthase large subunit
coaA	AB863013.1	F			1.6		-3.2				pantothenate kinase
dukA	GEB08937.1	F		-1.7	-1.8		-2.2				deoxynucleoside kinase nucleotide and nucleoside interconversions
gmk	NC_022369.1	F	-2.0								guanylate kinase
nrdE	NZ_CP094882.1	F	-2.1					-1.5			ribonucleotide-diphosphate reductase subunit alpha
nrdG	NC_020450.1	F	-1.6								anaerobic ribonucleoside-triphosphate reductase

pbuX	NC_000964.3	F	-1.8								xanthine/uracil permease
purR	NP_268416.1	F	1.9			1.7					*pur* operon repressor general regulatory function
pydB	NP_267502.1	F			-2.5	-1.5	-2.4				dihydroorotate transferase
pyrB	NP_267758.1	F			-2.1						aspartate carbamoyltransferase catalytic subunit
pyrC	NP_267225.1	F			-2.2		-1.8				dihydroorotase
pyrE	NP_267224.1	F			-2.7	-2.0	-2.2		-1.9		orotate phosphoribosyltransferase
pyrF	NP_267501.1	F			-1.9		-1.8				orotidine 5'-phosphate decarboxylase type 1 subfamily
pyrP	NP_267759.1	F			-2.6		-2.1				uracil permease
pyrR	SPS10181.1	F					-2.4				bifunctional pyrimidine regulatory protein PyrR
xpt	AAK05244.1	F	-2.8								xanthine phosphoribosyltransferase
yfiC	NP_266745.1	F		-1.6							hypothetical protein
yfiG	NP_266749.1	F	-2.0								thymidine kinase
ygcC	NP_266791.1	F	-1.7								hypothetical protein
yriD	NP_267887.1	F	-1.7								xanthine/uracil/vitamin C permease
**Carbohydrate transport and metabolism**											
agl	NP_267834.1	G							4.8		alpha-glucosidase degradation of polysaccharides
amyL	AE005176.1	G				1.7					alpha-amylase
amyY	AE005176.1	G							5.1		alpha-amylase
dexA	AE005176.1	G							4.7		oligo—alpha-1,6-glucosidase
dexC	GFO79576.1	G							5.1		neopullulanase
dhaL	NC_022369.1	G				1.6				1.9	dihydroxyacetone kinase
dhaM	NC_022369.1	G				1.5				1.6	dihydroxyacetone kinase
eno	NC_022369.1	G				2.3			3.9		phosphopyruvate hydratase enolase
fruA	AAK05057.1	G	2.0			1.8					PTS system fructose-specific transporter subunit IIABC
fruC	NC_015902.1	G	2.1		1.7	2.2					1-phosphofructokinase
galE	WP_058218180.1	G		-1.9			-1.9				UDP-glucose 4-epimerase
galK	NC_002662.1	G		-3.4	-3.7		-3.6	-2.9	-2.4	-3.2	galactokinase

galM	NC_020450.1	G		-3.4	-3.5		-3.7	-2.9	-2.3	-2.9	aldose 1-epimerase
galT	NC_022369.1	G		-3.6	-3.9		-3.3	-3.2	-3.0	-2.6	galactose-1-phosphate uridylyltransferase
galP	WP_004254704.1	G		-3.7	-4.0		-3.0	-3.0	-2.4	-2.5	glycoside-pentoside-hexuronide (GHP): cation symporter
thgA	NP_268138.1	G		-2.9	-3.0		-3.0	-2.4	-2.0	-2.4	thiogalactoside acetyltransferase
lacZ	NC_002662.1	G		-3.1	-3.5		-3.4	-2.8	-2.4	-3.1	beta-D-galactosidase
gapA	NC_020450.1	G	2.6								glyceraldehyde 3-phosphate dehydrogenase
glpF1	NC_009004.1	G	1.7			1.7					glycerol uptake facilitator
glpT	NC_009004.1	G							1.5		glycerol-3-phosphatase transporter
kdgA	NC_002662.1	G		2.1	1.8				2.4		keto-hydroxyglutarate-aldolase
malE	AAK05781.1	G								-5.4	maltose ABC transporter substrate binding protein
malF	AAK05782.1	G								-5.3	maltose ABC transporter permease
malG	AAK05783.1	G								-4.5	maltose ABC transporter permease
malQ	NC_002662.1	G				1.6					4-alpha-glucanotransferase
mtlF	NC_020450.1	G							1.6		PTS system mannitol-specific transporter subunit IIA
nagA	NZ_CP094882.1	G		-1.5							N-acetylglucosamine-6-phosphate deacetylase
ptbA	NP_267616.1	G								-1.6	PTS system beta-glucoside-specific transporter subunit IIABC
rpiA	NP_268390.1	G	2.0			1.7				2.1	ribose-5-phosphate isomerase A
tagH	NP_267071.1	G		1.6	1.6				1.9		teichoic acid ABC transporter ATP binding protein
xylA	NP_267665.1	G				2.0					xylose isomerase
xylH	NP_266730.1	G					-1.6				4-oxalocrotonate tautomerase
xylT	AAK05601.1	G		2.1	2.1				2.4		D-xylose proton-symporter
xynB	NP_267664.1	G							1.6		beta-1,4-xylosidase
xynT	AAK05604.1	G			1.6				2.1		xyloside transporter
ycgC	NP_266420.1	G	1.5								hypothetical protein
yeeB	AAK04528.1	G	2.1								hydrolase
yfbG	NP_266680.1	G			-5.4						hypothetical protein
yhgD	NP_266913.1	G		2.7	2.6				3.2		hypothetical protein

yhgE	AAK04856.1	G		4.1	3.7		2.3		4.0		hypothetical protein
yoiC	NP_267614.1	G							1.6		hypothetical protein
ypcA	NP_267645.1	G			2.3				2.4		P-beta-glucosidase
ypcC	NP_267647.1	G			-1.6						hypothetical protein
ypcD	NP_267648.1	G			-1.6						endo-beta-N-acetylglucosaminidase
ypjA	NP_267705.1	G	1.9	1.5	1.7	2.4					dehydrogenase
yqgE	NP_267779.1	G			1.8						transporter
yqhA	AAK05727.1	G			-1.7						hypothetical protein ∣ aldose-1-epimerase
yvdD	NP_268205.1	G			1.5						transporter
yxdE	NP_268407.1	G		1.6							oxidoreductase
yxdG	NP_268409.1	G		2.6	2.7	2.7	1.9	2.0	3.3		transporter
yxfA	NP_268421.1	G							-1.7		transporter
**Coenzyme transport and metabolism**											
apbE	AE005176.1	H							-2.3		thiamine biosynthesis lipoprotein
hemH	NC_022369.1	H				-1.7					ferrochelatase protoheme ferro-lyase
nadE	NC_022369.1	H	3.2			1.9					NAD synthetase
pabA	NC_000913.3	H				-2.2			-2.4		para-aminobenzoate synthase component II folic acid
pabB	NC_000913.3	H				-2.5			-2.4	-1.7	para-aminobenzoate synthase component I folic acid
preA	NC_013656.1	H		1.5	1.5		1.9				prenyl transferase
ribG	NP_267149.1	H			1.6		1.9				riboflavin-specific deaminase riboflavin and cobalamin
**Lipid transport and metabolism**											
accB	WP_012897457.1	I							2.4		acetyl-CoA carboxylase biotin carboxyl carrier protein subunit
accC	WP_012897458.1	I							1.7		acetyl-CoA carboxylase biotin carboxylase subunit
accD	SPS12911.1	I							1.6		acetyl-CoA carboxylase subunit beta
acpD	CP009472.1	I			-1.9						ACP phosphodiesterase
butA	NC_020450.1	I				1.5					acetoin reductase fermentation
fabD	NC_022369.1	I							2.3		ACP S-malonyltransferase

fabI	NC_022369.1	I			1.7						enoyl-ACP reductase
fabZ	NC_022369.1	I			1.6				2.2		(3R)-hydroxymyristoyl-ACP dehydratase
fadD	AE005176.1	I				-5.9			2.1		long-chain acyl-CoA synthetase
yfbB	NP_461239.3	I			-7.2		-1.8			-2.4	hypothetical protein
ygbB	NP_266781.1	I		1.7	2.2	1.7	1.5				hypothetical protein
yqaG	NP_267722.1	I			-2.7	-1.5					hypothetical protein
yveB	NP_268212.1	I							1.6		hypothetical protein
yveH	NP_268218.1	I			4.0				1.6		hypothetical protein
**Translation, ribosomal structure and biogenesis**											
aldR	JXKB01000001.1	J	1.5								AldR regulatory protein
rluC	NP_267508.1	J								1.7	pseudouridine synthase
truB	NP_267282.1	J							-1.6		tRNA pseudouridine synthase B
yabC	NP_266164.1	J		1.8							hypothetical protein
ybiD	NP_266335.1	J				1.5					ribosomal RNA large subunit methyltransferase
ygdA	NP_266799.1	J			1.6	1.9				1.7	hypothetical protein
ynjI	AAK05309.1	J	1.9				1.7	1.6			hypothetical protein
ynjJ	NP_267518.1	J	2.3				1.6				hypothetical protein
ywfF	NP_268336.1	J	-1.7								hypothetical protein
ywfG	NP_268337.1	J	2.4			2.5					hypothetical protein
**Transcription**											
adaA	WP_129881369.1	K							1.9		methylphosphotriester-DNA alkyltransferase
cspD	AE005176.1	K			-6.1						cold shock protein D
cspE	Y17217.1	K			1.6						cold shock protein E
dhaK	NP_266402.2	K				2.0			2.3	1.9	*dhaKLM* operon coactivator, dihydroxyacetone utilization pathway
fur	NC_002662.1	K	2.2								ferric uptake regulator
gadR	NZ_CP017194.1	K		-2.2			-1.9				positive regulator
hrcA	NC_022369.1	K	1.6								heat-inducible transcription repressor

fruR	NC_017478.1	K				1.6				DeoR family transcription regulator of fructose utilization pathway
nadR	NC_020450.1	K	2.1			1.7				NadR family transcription regulator, NAD cellular biosynthesis pathway
phoU	NC_022369.1	K	2.1							phosphate transport system regulator
rcfB	NP_268391.1	K				1.8				CRP/FNR family transcription regulator involved in pH homeostasis
rliA	NP_267832.1	K	2.8	1.6					1.9	transcriptional regulator LacI-family regulators, putative regulatory function
rliDB	NP_268306.1	K		3.0	2.3	3.2	2.1		3.3	transcriptional regulator LacI-family regulators, putative regulatory function
rlrA	NP_267392.1	K							-1.6	LysR-family transcription regulator, putative regulatory function
rmaA	NP_266898.1	K		-1.6		-1.6				MarR family transcriptional regulator of fatty acid biosynthesis pathway
rmaG	NP_266926.1	K		-2.1						MarR family transcriptional regulator of fatty acid biosynthesis pathway
rmaJ	NP_266747.1	K	-1.6							MarR-family transcriptional regulator of hyaluronan biosynthesis pathway
rpoD	NP_266709.1	K	1.8							RNA polymerase sigma factor RpoD
sigX	NP_268323.1	K							1.6	RNA polymerase ECF sigma factor
tagR	NP_267067.1	K							1.9	transcriptional regulator similar to EpsR, possibly involved in exopolysaccharides biosynthesis
ybaI	NP_266263.1	K	-1.8							glycosyl transferase glycosyltransferase family 2
ybeF	NP_266307.1	K		1.7	2.0	1.9			2.3	hypothetical protein
yfeA	NP_266710.1	K		1.6		2.1				RpiR family of transcription regulator, putative regulatory function
ygfC	NP_266817.1	K		-2.9		-4.8				TetR/AcrR family transcription regulator of heme homeostastis processess
yjaB	NP_267035.1	K	2.5							transcriptional regulator, LytR family, putative regulatory function

yjaJ	AAK04985.1	K		1.6							transcriptional regulator, LytR family, putative regulatory function
ykhD	NP_267216.1	K			1.8						redox-sensing transcriptional repressor Rex
yliA	NP_267323.1	K	2.0								positive transcription regulator
ynaA	NP_267426.1	K	2.2								transcriptional regulator, heat and DNA damage induction
ynaB	AAK05369.1	K	2.2							1.5	transcriptional regulator, putative regulatory function
ynaE	NP_267430.2	K			1.6						hypothetical protein, putative regulatory function
yogM	NP_267604.1	K		1.6	1.6						hypothetical protein
yohC	AAK05547.1	K						1.6			TetR/AcrR family transcription regulator, putative regulatory function
yqfA	NP_266815.1	K			-1.7						hypothetical protein
yrfA	NP_267850.1	K	1.5								ArsR family transcription regulator, putative regulatory function
yrfE	NP_267858.1	K		-1.6							LytR family transcriptional regulator , putative regulatory function
glaR	NP_268142.1	K		-1.8				-1.7			RpiR family transcription regulator of galactose utilization Leloir pathway
ywjD	NP_268370.1	K		-1.7		-1.7			-1.8		PBSX(XRE) family transcription regulator, putative regulatory function
yxbF	NP_268394.1	K		-2.2			2.4				hypothetical protein
**Replication, recombination and repair**											
comEA	NC_002662.1	L		1.6							hypothetical protein competence protein
dinG	NC_022369.1	L				-1.5					DinG ATP-dependent helicase
dinP	NC_000962.3	L							1.7		DNA polymerase IV
hslB	NC_002662.1	L	2.5							2.3	HU-like DNA-binding protein
recJ	SPS10369.1	L	-1.6								ssDNA-specific exonuclease RecJ
recX	NP_268339.1	L	1.7								recombination regulator RecX
ssbA	NP_266548.1	L				3.1			1.6		single-stranded DNA-binding protein
xerD	NP_266801.1	L		1.5							integrase-recombinase

yajF	NP_266245.1	L				2.4			1.7		hypothetical protein
yjaF	NP_267039.1	L	2.4								hypothetical protein
yneB	NP_267463.1	L				1.8					hypothetical protein
**Cell wall, membrane and envelope biogenesis**											
dapA	SPS10159.1	M	1.6								dihydrodipicolinate synthase
dltB	NC_022369.1	M	2.8	-1.7		2.1		-1.6	-1.7	-2.4	peptidoglycan biosynthesis protein
dltD	NC_022369.1	M	4.1	-1.6		3.6					peptidoglycan biosynthesis protein
glmS	WP_058223713.1	M	1.8								glucosamine--fructose-6-phosphate aminotransferase
mscL	NZ_CP094882.1	M	1.8								large-conductance mechanosensitive channel
murB	SPS10276.1	M			1.9						UDP-N-acetylenolpyruvoylglucosamine reductase
murQ	NZ_CP094882.1	M		-1.8					-1.6		N-acetylmuramic acid 6-phosphate etherase
plpA	NC_002662.1	M	-2.8	-2.2	-1.6	-3.0	-1.8	-1.6	-2.5		outer membrane lipoprotein
plpB	AAK04417.1	M				-2.1			-1.7		outer membrane lipoprotein
plpC	NC_002662.1	M		-1.6		-2.4			-1.8		outer membrane lipoprotein
ptk	NP_267658.1	M	2.7			1.9					phosphoketolase
tagL	NP_267068.1	M		2.0							exopolysaccharide biosynthesis protein
ycbH	NP_266371.1	M	-1.6								sugar transferase
ycbI	NP_266372.1	M	-1.7								sugar transferase
ycbK	NP_266368.1	M	-1.7								polysaccharide biosynthesis export protein
yfbI	NP_266682.1	M			-7.6						hypothetical protein
yfbJ	NP_266683.1	M			-7.2						hypothetical protein
yijB	NP_267025.1	M			1.7						hypothetical protein
yijC	NP_267026.1	M			1.7						ABC transporter permease transport and binding protein
yijH	NP_267033.1	M	1.8								hypothetical protein
ymjE	NP_267421.1	M	1.5								glycosyl transferase family protein
yncA	NP_267443.1	M				1.7					acetyltransferase
ynfC	NP_267475.1	M	1.5								hypothetical protein
ytbA	NP_268002.1	M		1.6					2.1		hypothetical protein
ytjA	NP_268069.1	M	1.8								hypothetical protein

**Posttranslational modification, protein turnover, chaperones**											
clpE	AF023421.1	O	2.4								ATP-dependent protease
gpo	NC_020450.1	O	1.8								glutathione peroxidase thioredoxin, glutaredoxin, and glutathione
groES	NC_022369.1	O	2.8								co-chaperonin GroES 10 kDa chaperonin
osmC	PLW60135.1	O	2.5								osmotically inducible protein
pmsX	NC_020450.1	O	2.0								peptide methionine sulfoxide reductase t
sugE	NP_266278.1	O		1.8	2.3	2.2					SugE protein chaperones
ybjA	NP_266345.1	O	1.9								methionine sulfoxide reductase B
yfcF	NP_266694.1	O		2.0	1.5	2.0			1.9		hypothetical protein
ynhC	NP_267492.1	O	1.5								hypothetical protein
ynhD	NP_267493.1	O			1.7						hypothetical protein
**Inorganic ion transport and metabolism**											
amtB	AAK05690.1	P				-1.7		1.9	-1.7	-2.2	ammonium transporter
busAB	AF393650.1	P					1.8				betaine ABC transporter permease and substrate binding protein
cadA	U78967.1	P	2.3			3.7			2.4		cadmium efflux ATPase transort and binding protein
cbiO	AE005176.1	P				2.3			3.9		cobalt transporter ATP-binding subunit
feoA	NC_022369.1	P								1.5	ferrous ion transport protein A
mtsA	AAK05414.1	P				-3.2			-3.7	-3.6	manganese ABC importer
mtsB	AAK05416.1	P				-2.0			-2.7		manganese ABC importer
mtsC	AAK05415.1	P				-2.0			-2.6	-3.8	manganese ABC importer permease
pacL	AAK04776.1	P				2.3			2.4		magnesium-transporting ATPase
pstF	NC_022369.1	P	1.5								phosphate ABC transporter substrate-binding protein
yafB	AAK04148.1	P		2.1	1.6	2.7			2.6		sulfate permease, MFS superfamily
ybcC	NP_266275.1	P		1.8		1.7			1.7		MATE family efflux transporter
yceA	NP_266398.1	P		1.6					1.6		hypothetical protein
ydiF	NP_266539.1	P					1.7				Na^+/H^+ antiporter transort and binding protein, cations
ygfE	AAK04761.1	P			1.8	-5.7		-1.9	-2.7	2.0	magnesium importer and exporter

yjdJ	NP_266539.1	P		1.7		2.1			1.8		potassium channel protein transort and binding protein, cations
ymgF	NP_267394.1	P		1.9			1.6				hypothetical protein
ynjC	NP_267510.1	P							1.6		hypothetical protein
ynjE	NP_267512.1	P		1.9	1.5				1.6		hypothetical protein
ynjF	NP_267513.1	P							1.6		hypothetical protein
ypbB	NP_267639.1	P		1.8		2.0			2.1		magnesium transporter transport and binding protein
ytgB	NP_268049.1	P	2.3			2.0					cesium ABC transporter substrate binding protein
yuhE	AAK06092.1	P	2.5			1.5				1.8	copper homeostasis protein
yvfA	NP_268222.1	P		1.5							metal ABC transporter substrate binding protein
**Secondary metabolites biosynthesis, transport and catabolism**											
aldC	NC_000962.3	Q			2.0						alpha-acetolactate decarboxylase fermentation
dltA	WP_058205708.1	Q	3.0			2.1					D-alanine-poly(phosphoribitol) ligase subunit 1
dltC	QQE99144.1	Q		-1.9		2.2			-1.8	-2.2	D-alanine-poly(phosphoribitol) ligase subunit 2
fabG2	AE005176.1	Q		2.1	1.9	2.1	1.6		2.6		3-oxoacyl-ACP reductase
yoaI	NP_267527.1	Q				1.6					hypothetical protein
yrbC	NP_267819.1	Q							1.8		1,4-dihydroxy-2-naphthoate octaprenyltransferase
yrbE	NP_267821.1	Q			1.6				2.0		hypothetical protein
**General function prediction**											
comEC	AAK05870.1	R		1.9	1.9				2.4		hypothetical protein competence protein
yhhD	AAK04860.1	R		2.0	1.7		1.5		2.6		hypothetical protein
ysbA	AAK05851.1	R		1.6					1.6		hypothetical protein
ysbD	NP_267914.1	R	-2.2					-1.8			hypothetical protein
ythC	AAK06004.1	R			2.4						hypothetical protein
**Function unknown**											
L200065	YP_005869326.1	S							2.3	1.7	hypothetical protein
yafJ	NP_266211.1	S		1.6					2.0		hypothetical protein
yaiB	NP_266234.1	S		1.7		1.7			2.0		hypothetical protein

yaiH	NP_266241.1	S			1.7		1.6	1.7			hypothetical protein
yaiI	NP_266239.1	S		2.4	2.0				2.4		hypothetical protein
yaiJ	BAL52160.1	S		2.6	2.2		1.8		3.2		hypothetical protein
ybeH	NP_266301.1	S				2.3			1.9		hypothetical protein
ybfC	NP_266313.1	S			2.0						hypothetical protein
ybgA	NP_266314.1	S		1.6	1.8						hypothetical protein
ycdA	NP_266387.1	S			1.6						hypothetical protein
yciF	NP_266444.1	S		1.5							hypothetical protein
yciG	NP_266445.1	S		1.8		2.1			2.3		hypothetical protein
ydbD	AAK04412.1	S							1.5		hypothetical protein
yeiD	NP_266641.1	S	2.2				-2.3				hypothetical protein
yfbK	NP_266684.1	S			-3.5		-2.1				hypothetical protein
yfiH	AAK04692.1	S	-1.8								hypothetical protein
yfiI	AAK04694.1	S	-2.0								hypothetical protein
ygaD	AAK04713.1	S	-1.8								hypothetical protein
ygeB	NP_266810.1	S				-1.6			-2.1		hypothetical protein
ygeC	NP_266811.1	S	-2.1								hypothetical protein
yghB	NP_266830.1	S						1.5			hypothetical protein
yhhA	NP_266915.1	S		1.8					2.3		hypothetical protein
yhhB	NP_266916.1	S							1.7		hypothetical protein
yhhC	NP_266917.1	S				2.6					hypothetical protein
yhjA	AAK04881.1	S	4.3		2.1	1.9				2.5	hypothetical protein
yhjE	NP_266943.1	S							-1.7		hypothetical protein
yiaC	NP_266951.1	S	-1.8								hypothetical protein
yiiD	NP_267017.1	S				2.1					hypothetical protein
yjdI	NP_267069.1	S		2.0					1.8		hypothetical protein
yjfJ	NP_267090.1	S	1.6								hypothetical protein
ykbC	AAK05084.1	S		1.8					2.2		hypothetical protein
ykbD	NP_267143.1	S		1.9	1.5				1.9		hypothetical protein

ykbE	NP_267144.1	S				1.6				hypothetical protein
ykcE	NP_267155.1	S	2.0							hypothetical protein
ykjI	NP_267241.1	S		-2.0					-2.3	hypothetical protein
ylhB	NP_267317.1	S			2.5					hypothetical protein
yliD	AAK05268.1	S		2.5	1.9				2.6	hypothetical protein
ymbC	NP_267348.1	S			1.7	2.0		1.9	1.9	hypothetical protein
ymbD	NP_267349.1	S		2.3	2.0	2.1			2.6	hypothetical protein
ymbH	NP_835314.1	S		1.7					1.8	hypothetical protein, putative protease
ymcA	NP_267355.1	S		1.6					2.1	hypothetical protein
ymcB	NP_267356.1	S		2.0					2.0	hypothetical protein
ymcC	NP_267357.1	S		2.2					1.6	hypothetical protein
ymeA	AAK05317.1	S		2.6	2.5		1.8		3.2	hypothetical protein
ymhG	YP_007508531.1	S							1.8	hypothetical protein
yndE	NP_267456.1	S		1.7						hypothetical protein
yneC	NP_267464.1	S	4.0		2.4	3.1				hypothetical protein
yneG	NP_267468.1	S			2.6					hypothetical protein
ynfH	NP_267481.1	S		2.4		2.7	2.9	1.9	2.1	hypothetical protein
ynhA	NP_267490.1	S							-2.8	hypothetical protein
yniC	NP_267500.1	S					-1.6			hypothetical protein
yniJ	NP_267507.1	S		1.7		1.7			2.1	hypothetical protein
yofM	NP_267590.1	S		-2.0					-1.8	DNA-binding protein
yohD	NP_267606.1	S	1.7							hypothetical protein
ypaG	NP_267633.1	S				1.6				hypothetical protein
ypiH	AAK05645.1	S							1.7	hypothetical protein
ypiJ	NP_267702.1	S							1.8	hypothetical protein
ypiL	NP_267704.1	S				1.5				hypothetical protein
yqbI	YP_007508825.1	S			1.6				2.0	hypothetical protein
yraE	NP_267813.1	S						1.6		hypothetical protein
yrbB	AAK05760.1	S					-1.7			hypothetical protein

yrbH	AAK05766.1	S		1.8	1.6				2.6		hypothetical protein
yreB	NP_267843.1	S	-1.6								hypothetical protein
yreD	AAK05787.1	S		3.7					3.9	3.8	hypothetical protein
yseD	AAK05883.1	S		2.0		2.0			2.3		hypothetical protein
ysjF	AAK05925.1	S		1.8		2.0			2.6		hypothetical protein
ytbD	NP_268007.1	S	3.3								hypothetical protein
ytcD	NP_268015.1	S	2.3								hypothetical protein
yuaE	NP_268084.2	S						-2.1	-1.9		hypothetical protein
yueC	NP_268082.1	S	1.8	2.0	1.8				2.0		hypothetical protein
yuhC	NP_268149.1	S	1.5								hypothetical protein
yujF	NP_268177.1	S		2.4	1.9				2.3		hypothetical protein
yviJ	NP_268276.1	S		2.0	1.8	2.9					hypothetical protein
yxcD	NP_268398.1	S			1.9					1.5	hypothetical protein
**Signal transduction mechanisms**											
floL	NC_002662.1	T	1.6								flotillin-like protein
kinA	NC_002662.1	T				-1.7					sensor protein kinase
kinB	NC_002662.1	T	1.7								sensor protein kinase
llrA	NC_019435.1	T	1.7								two-component system regulator
ybdC	NP_266285.1	T	-2.1			-1.5					hypothetical protein
yfgF	NP_266724.1	T							2.0		ABC transporter permease
yjjE	NP_267123.1	T	2.6								hypothetical protein
ythA	AAK06002.1	T	1.9		3.3						hypothetical protein
ythB	AAK06003.1	T			2.8						hypothetical protein
yudH	NP_268117.1	T		-1.7							hypothetical protein dihydrofolate reductase
**Intracellular trafficking, secretion, and vesicular transport**											
comC	NC_002662.1	U							2.0		type 4 prepilin-like protein specific leader peptidase transformation
comGA	AAK06221.1	U		2.6	2.6				2.8		protein ComGA competence protein
comGB	NC_000964.3	U		2.5	1.8				2.6		protein ComGB competence protein

comGC	NC_022369.1	U		2.2		2.6			2.6		protein ComGC competence protein
comGD	NZ_CP059048.1	U		2.7	1.7		1.5	1.9	1.6		protein ComGD competence protein
ftsY	NC_022369.1	U	1.7		1.7						hypothetical protein
**Defence mechanisms**											
lmrP	QQF00039.1	V	-1.9			2.5	1.5		1.8		multidrug efflux MFS transporter
ybdI	NP_266290.1	V					1.6	1.7			hypothetical protein
ybfD	NP_266310.1	V		1.6		2.4			1.9		transporter transport and binding protein
yfjF	NP_266765.1	V				2.7			1.9		membrane transport protein transport and binding protein
ygfA	NP_266815.1	V				-2.8					ABC transporter ATP-binding protein
ygfB	NP_266816.1	V				-4.8					ABC transporter permease
yhcA	NP_266870.1	V			1.5						ABC transporter ATP-binding protein/permease
yhcC	NP_266871.1	V				2.0					hypothetical protein
yhcE	NP_266874.1	V		-2.1					-1.7		hypothetical protein
ykjJ	NP_267248.1	V			1.5				1.8		hypothetical protein
ymdC	AAK05309.1	V							1.8		kanamycin kinase
ypbC	AAK05582.1	V		2.2	1.9				2.8		MATE family efflux transporter
ypfE	NP_267672.1	V		1.8	1.9				2.0		transporter transport and binding protein
ypgD	AAK05620.1	V		-1.7							multidrug resistance ABC transporter ATP-binding protein
yvhA	AAK06200.1	V	1.7			1.6					MATE family efflux transporter
yweA	NP_268321.1	V		1.7					2.0		membrane protein
yweE	NP_268328.1	V	2.1			1.5					hypothetical protein
yweF	NP_268329.1	V	1.8								hypothetical protein
ywiG	AAK06301.1	V		2.5	1.8				2.8		ABC-type multidrug transport system, ATPase component
yxbD	NP_268392.1	V				3.9	3.1			-3.0	multidrug transporter transport and binding protein
yxbE	NP_268393.1	V		-2.2			2.2			-2.1	hypothetical protein
yxeA	NP_268412.1	V		1.6			1.9				hypothetical protein

Table S2. Transcriptomic data set with relaxed Log2Ratio thresholds. A gene was identified as differentially expressed between the *L. lactis* strains if its transcript level differed by|Log2Ratio|≥1.5 with statistical significance, $p<0.05$. Bold and italic font shows chromosomal genes differentially expressed in the presence of plasmids with transcript levels 20% below the applied significance threshold (|Log2Ratio|≥1.2) with statistical significance, p<0.05.

Gene	Gene upregulation or downregulation *L. lactis* strain								Protein function
	**IL1618**	**IL1421**	**IL2661**	**IL1619**	**IL1420**	**IL1530**	**IL1392**	**IL594**	
Plasmid / plasmids harboured	**pIL1**	**pIL3**	**pIL4**	**pIL5**	**pIL6**	**pIL7**	**pIL1, pIL2, pIL3, pIL5**	**pIL1-pIL7**	
adhE	***1.3***		3.2		1.7	2.1	2.2		bifunctional acetaldehyde-CoA
agl							4.8		alpha-glucosidase
citB		1.8		2.0		2.3	2.5		acetate-SH-citrate lyase
citC							2.3		acetate-SH-citrate lyase
citD							3.0		citrate lyase subunit gamma
gltA				2.1		1.6			citrate synthase
gltP		1.8		1.5	3.0		2.1		glutamate ABC transporter permease
gltQ					2.9	1.8	1.9		glutamate ABC transporter ATP-binding protein
gltS		1.9		2.3	3.2		3.0		glutamate or arginine ABC transporter substrate binding protein
ipd				1.6			***1.5***		indole-3-pyruvate decarboxylase
maa							4.6		maltose O-acetyltransferase
mleP		3.0					3.2		malate transporter
mleS		3.0					2.9		malate dehydrogenase
msmK								-2.6	multiple sugar ABC transporter ATP-binding protein
pdhB							2.1		PDH E1 component subunit beta
pydA			1.8				***1.3***		dihydroorotate dehydrogenase 1A
pyrZ			-2.0		-2.4				dihydroorotate dehydrogenase electron transfer subunit
trxA	2.6			***1.4***					thioredoxin
trxB1	1.8								thioredoxin reductase

tyrS	-1.7			-2.5					tyrosyl-tRNA synthetase
ybiE	1.9						3.3		oxidoreductase
yddB		***1.3***					2.0		oxidoreductase
yfdE	-1.8								hypothetical protein similar to myo-inositol monophosphatases
yfhC	2.6		1.5	3.7					hypothetical protein
ygaJ	2.0		***1.2***						putative peptidase
yiaD	3.4		1.9	***1.4***			2.1		NADH-flavin reductase
yjcA		1.7	***-1.2***						ABC transporter ATP-binding protein
yjgC		***-1.4***						-1.5	amino acid ABC transporter substrate binding protein
ylbA	1.6								ABC transporter ATP-binding protein
ymgG	***1.4***	1.7		***1.5***	***1.4***				hypothetical protein
ymgH		1.6			***1.4***	1.5			hypothetical protein
ymgK	1.5						2.4		oxidoreductase
yobA	1.9		***1.4***	1.7					hypothetical protein
yogA					-1.7				hypothetical protein
yphC					-3.9				oxidoreductase
yqcA		***1.2***					1.7		oxidoreductase
yrjA	-1.5								hypothetical protein
yrjB	***1.4***	1.6	1.5		2.0	1.9			oxidoreductase
yrjC					1.7				iron-binding oxidase subunit
yugB				***1.5***			2.3		oxidoreductase
ywdA		***1.3***					1.8		hypothetical protein
ywdC		1.7					1.7		hypothetical protein
ywiD	2.9								hypothetical protein
ywiH		2.8	2.2		1.6		3.3		ABC transporter permease
ywjF				1.6			***1.4***		3-hydroxyisobutyrate dehydrogenase
ezrA	2.4			1.8					septation ring formation regulator EzrA
scpA							-1.6		segregation and condensation protein A
yihA		1.8	1.8				1.8		hypothetical protein

yihB		1.7	1.6				1.5		hypothetical protein
arcA	1.8				3.0	2.8	2.5		arginine deiminase
arcB	***1.4***	1.7			2.8	2.2	2.3		ornithine carbamoyltransferase
arcC1					2.7				carbamate kinase
arcC2	2.6	1.7			2.6		***1.4***		carbamate kinase
arcD1		1.8			3.2	1.9	2.1		arginine/ornitine antiporter
argF	***1.3***	1.7			***1.3***		1.7		ornithine carbamoyltransferase
aroH				-2.0					phospho-2-dehydro-3-deoxyheptonate aldolase
asd							2.3		aspartate-semialdehyde dehydrogenase
bmpA							-2.2		basic membrane protein A
ceo					1.7				N5-carboxyethyl-ornithine synthase
cysD							-2.2		O-acetylhomoserine sulfhydrylase
cysK		-2.5	***-1.4***		-2.6	-1.6	-2.7		cysteine synthase serine family
cysM		-3.0			-2.9	-1.7	-2.4		cysteine synthase
glnB						2.1			nitrogen regulatory protein P-II
glnQ			***-1.4***					-1.9	glutamine ABC transporter ATP-binding protein
hisA						1.5			1-(5-phosphoribosyl)-5-[(5-phosphoribosylamino)methylideneamino] imidazole-4-carboxamide isomerase
ilvD		***1.5***	***1.3***				2.2		dihydroxy-acid dehydratase
leuC						1.5	***1.4***		isopropylmalate isomerase large subunit dehydratase component
lysP		2.0	***1.2***		***1.3***		2.3		lysine specific permease
mapA	2.8						5.2		maltose phosphorylase
metB1	-1.5	***-1.5***		***-1.3***					cystathionine gamma-synthase
metB2		-2.8	-1.6		-2.4	-1.6	-3.2		cystathionine gamma-synthase
oppA		1.8	***1.4***				2.4		oligopeptide ABC transporter substrate binding protein
oppB		3.2	7.6		***1.5***		3.8	6.4	oligopeptide ABC transporter permease
oppC		***1.5***	7.9	2.0			2.0	6.3	oligopeptide ABC transporter permease
oppD			5.9					5.0	oligopeptide ABC transporter ATP binding protein
oppF		1.5	6.9				1.8	5.7	oligopeptide ABC transporter ATP binding protein

optS		1.8	***1.3***	2.3	***1.5***	1.5	2.2		oligopeptide ABC transporter substrate binding protein
pepDB	1.7								dipeptidase
pepF							2.0	1.6	oligoendopeptidase F
potA		***-1.3***	***-1.3***				-1.8		spermidine/putrescine ABC transporter ATP-binding protein
poxL	2.8	1.7	1.8	2.7			2.3		pyruvate oxidase
serB			1.6						phosphoserine phosphatase
ybeC	1.9		***1.3***					1.6	hypothetical protein
yceJ				1.6				***1.5***	hypothetical protein
ycjI			***-1.3***				-1.9		hypothetical protein
yddA		2.4	2.6				3.0		transporter transport and binding protein
ydgC							1.8		amino acid permease
yibG				-1.6					amino acid permease transport and binding protein
ylcC	2.0								hypothetical protein
ylcF		2.0	***1.4***				1.6		hypothetical protein
yneH			2.5						hypothetical protein
yrfB			***1.4***				1.8		NADH-dependent oxidoreductase
yrfD							1.8		amino acid antiporter
ysaB	3.7			3.0			2.2		ABC transporter permease and substrate binding protein
ysaC	3.0			2.8			2.1		ABC transporter ATP-binding protein
ysaD	2.8			2.9			1.6		hypothetical protein
yshA	***-1.4***			-2.1		-1.6			glutamate antiporter
ysjA							-2.2	-1.6	amino acid permease transport and binding protein
apt	-2.5								adenine phosphoribosyltransferase
carA			-2.3		-1.7				carbamoyl phosphate synthase small subunit
carB			-1.9		-2.3				carbamoyl phosphate synthase large subunit
coaA			1.6	***1.4***	-3.2				pantothenate kinase
dukA		-1.7	-1.8		-2.2				deoxynucleoside kinase nucleotide and nucleoside interconversions
gmk	-2.0								guanylate kinase
nrdE	-2.1					-1.5			ribonucleotide-diphosphate reductase subunit alpha

nrdG	-1.6					***-1.3***			anaerobic ribonucleoside-triphosphate reductase
pbuX	-1.8								xanthine/uracil permease
purR	1.9			1.7			***1.4***		pur operon repressor general regulatory function
pydB			-2.5	-1.5	-2.4		***-1.3***		dihydroorotate transferase
pyrB			-2.1						aspartate carbamoyltransferase catalytic subunit
pyrC			-2.2		-1.8		***-1.3***		dihydroorotase
pyrE			-2.7	-2.0	-2.2		-1.9		orotate phosphoribosyltransferase
pyrF			-1.9		-1.8				orotidine 5'-phosphate decarboxylase type 1 subfamily
pyrP			-2.6		-2.1		***-1.2***		uracil permease
pyrR			***-1.2***		-2.4				bifunctional pyrimidine regulatory protein PyrR
xpt	-2.8								xanthine phosphoribosyltransferase
yfiC		-1.6			***-1.4***				hypothetical protein
yfiG	-2.0			***-1.3***					thymidine kinase
ygcC	-1.7			***-1.3***					hypothetical protein
yriD	-1.7								xanthine/uracil/vitamin C permease
agl							4.8		alpha-glucosidase degradation of polysaccharides
amyL				1.7		***1.4***			alpha-amylase
amyY							5.1		alpha-amylase
dexA							4.7		oligo-alpha-1,6-glucosidase
dexC							5.1		neopullulanase
dhaL				1.6				1.9	dihydroxyacetone kinase subunit
dhaM				1.5				1.6	dihydroxyacetone kinase
eno				2.3			3.9		phosphopyruvate hydratase enolase
fruC	2.1		1.7	2.2					1-phosphofructokinase
fruA	2.0			1.8					PTS system fructose-specific transporter subunit IIABC
galE		-1.9	***-1.2***		-1.9	***-1.2***			UDP-glucose 4-epimerase
galK		-3.4	-3.7	***1.3***	-3.6	-2.9	-2.4	-3.2	galactokinase
galM		-3.4	-3.5	***1.4***	-3.7	-2.9	-2.3	-2.9	aldose 1-epimerase
galT		-3.6	-3.9		-3.3	-3.2	-3.0	-2.6	galactose-1-phosphate uridylyltransferase

gapA	2.6								glyceraldehyde 3-phosphate dehydrogenase
glpF1	1.7			1.7					glycerol uptake facilitator
glpT		***1.3***	***1.4***				1.5		glycerol-3-phosphatase transporter
kdgA		2.1	1.8				2.4		keto-hydroxyglutarate-aldolase
galP		-3.7	-4.0		-3.0	-3.0	-2.4	-2.5	glycoside-pentoside-hexuronide (GPH):cation symporter
lacZ		-3.1	-3.5		-3.4	-2.8	-2.4	-3.1	beta-D-galactosidase
malE								-5.4	maltose ABC transporter substrate binding protein
malF								-5.3	maltose ABC transporter permease
malG								-4.5	maltose ABC transporter permease
malQ				1.6			***1.3***		4-alpha-glucanotransferase
mtlF		***1.4***					1.6		PTS system mannitol-specific transporter subunit IIA
nagA		-1.5							N-acetylglucosamine-6-phosphate deacetylase
ptbA								-1.6	PTS system beta-glucoside-specific transporter subunit IIABC
rpiA	2.0			1.7				2.1	ribose-5-phosphate isomerase A
tagH		1.6	1.6				1.9		teichoic acid ABC transporter ATP binding protein
thgA		-2.9	-3.0		-3.0	-2.4	-2.0	-2.4	thiogalactoside acetyltransferase
xylA				2.0					xylose isomerase
xylH		***-1.4***			-1.6				4-oxalocrotonate tautomerase
xylT		2.1	2.1		***1.3***		2.4		D-xylose proton-symporter
xynB		***1.4***	***1.2***				1.6		beta-1,4-xylosidase
xynT			1.6				2.1		xyloside transporter
ycgC	1.5								hypothetical protein
yeeB	2.1								hydrolase
yfbG			-5.4						hypothetical protein
yhgD		2.7	2.6		***1.4***		3.2		hypothetical protein
yhgE		4.1	3.7		2.3		4.0		hypothetical protein
yoiC		***1.4***					1.6		hypothetical protein
ypcA			2.3				2.4		P-beta-glucosidase
ypcC			-1.6						hypothetical protein

ypcD			-1.6						endo-beta-N-acetylglucosaminidase
ypjA	1.9	1.5	1.7	2.4			***1.4***		dehydrogenase
yqgE	***1.4***	***1.4***	1.8	***1.5***					transporter
yqhA			-1.7	***-1.2***					hypothetical protein | aldose-1-epimerase
yvdD			1.5						transporter
yxdE		1.6							oxidoreductase
yxdG		2.6	2.7	2.7	1.9	2.0	3.3		transporter
yxfA							-1.7		transporter
apbE							-2.3		thiamine biosynthesis lipoprotein
hemH				-1.7					ferrochelatase protoheme ferro-lyase
nadE	3.2			1.9					NAD synthetase
pabA				-2.2			-2.4		para-aminobenzoate synthase component II folic acid
pabB				-2.5			-2.4	-1.7	para-aminobenzoate synthase component I folic acid
preA		1.5	1.5		1.9				prenyl transferase
ribG			1.6		1.9				riboflavin-specific deaminase riboflavin and cobalamin
accB			***-1.2***				2.4		acetyl-CoA carboxylase biotin carboxyl carrier protein subunit
accC							1.7		acetyl-CoA carboxylase biotin carboxylase subunit
accD							1.6		acetyl-CoA carboxylase subunit beta
acpD		***-1.5***	-1.9						ACP phosphodiesterase
butA	***1.3***			1.5					acetoin reductase fermentation
fabD							2.3		ACP S-malonyltransferase
fabI			1.7						enoyl-ACP reductase
fabZ							2.2		(3R)-hydroxymyristoyl-ACP dehydratase
fadD				-5.9			2.1		long-chain acyl-CoA synthetase
yfbB			-7.2		-1.8			-2.4	hypothetical protein
ygbB		1.7	2.2	1.7	1.5				hypothetical protein
yqaG		***-1.2***	-2.7	-1.5					hypothetical protein
yveB		***1.4***					1.6		hypothetical protein
yveH		***1.4***	4.0				1.6		hypothetical protein

aldR	1.5								AldR regulatory protein
rluC								1.7	pseudouridine synthase
truB				***-1.4***			-1.6		tRNA pseudouridine synthase B
yabC		1.8							hypothetical protein
ybiD	***1.3***			1.5					ribosomal RNA large subunit methyltransferase
ygdA			1.6	1.9			***1.2***	1.7	hypothetical protein
ynjI	1.9				1.7	1.6			hypothetical protein
ynjJ	2.3	***1.4***		***1.4***	1.6	***1.5***			hypothetical protein
ywfF	-1.7								hypothetical protein
ywfG	2.4	***1.2***		2.5			***1.3***		hypothetical protein
adaA							1.9		methylphosphotriester-DNA alkyltransferase
cspD			-6.1						cold shock protein D
cspE			1.6						cold shock protein E
dhaK				2.0			2.3	1.9	dhaKLM operon coactivator, dihydroxyacetone utilization pathway
fur	2.2								ferric uptake regulator
gadR		-2.2			-1.9				positive regulator
hrcA	1.6								heat-inducible transcription repressor
fruR				1.641					DeoR family transcription regulator of fructose utilization pathway
nadR	2.1	***1.5***		1.7					NadR family transcription regulator, NAD cellular biosynthesis patway
phoU	2.1			***1.3***					phosphate transport system regulator
rcfB				1.8					CRP/FNR family transcription regulator involved in pH homeostasis
rliA		1.6					1.9		transcriptional regulator LacI-family regulators, putative regulatory function
rliDB		3.0	2.3	3.2	2.1		3.3		transcriptional regulator LacI-family regulators, putative regulatory function
rlrA							-1.6		LysR-family transcription regulator, putative regulatory function
rmaA		-1.6	***-1.2***	-1.6					MarR family transcriptional regulator of fatty acid biosynthesis pathway
rmaG		-2.1							MarR family transcriptional regulator of fatty acid biosynthesis pathway
rmaJ	-1.6								MarR-family transcriptional regulator of hyaluronan biosynthesis pathway

rpoD	1.8								RNA polymerase sigma factor RpoD
sigX							1.6		RNA polymerase ECF sigma factor
tagR			***1.5***				1.9		transcriptional regulator similar to EpsR, possibly involved in exopolysaccharides biosynthesis
yabA									transcriptional regulator belongs to the PBSX(XRE) family of transcription regulators
ybaI	-1.8								glycosyl transferase glycosyltransferase family 2
ybeF		1.7	2.0	1.9			2.3		hypothetical protein
yfeA		1.6	***1.5***	2.1					RpiR family of transcription regulator, putative regulatory function
ygfC		-2.9		-4.8					TetR/AcrR family transcription regulator of heme homeostastis processess
yjaB	2.5								transcriptional regulator, LytR family, putative regulatory function
yjaJ		1.6							transcriptional regulator, LytR family, putative regulatory function
ykhD			1.8						redox-sensing transcriptional repressor Rex
yliA	2.0		***1.3***					***1.4***	positive transcription regulator
ynaA	2.2		***-1.5***						transcriptional regulator, heat and DNA damage induction
ynaB	2.2							1.5	transcriptional regulator, putative regulatory function
ynaE			1.6						hypothetical protein, putative regulatory function
yogM		1.6	1.6				***1.4***	***1.3***	hypothetical protein
yohC						1.6			TetR/AcrR family transcription regulator, putative regulatory function
yqfA		***-1.4***	-1.7				***-1.4***		hypothetical protein
yrfA	1.5		***1.4***						ArsR family transcription regulator, putative regulatory function
yrfE		-1.6							LytR family transcriptional regulator , putative regulatory function
glaR		-1.8				-1.7	***-1.3***		RpiR family transcription regulator of galactose utilization Leloir pathway
ywjD		-1.7		-1.7		***-1.4***	-1.8		PBSX(XRE) family transcription regulator, putative regulatory function
yxbF		-2.2			2.4				hypothetical protein
comEA		1.6							hypothetical protein competence protein
dinG		***-1.3***		-1.5			***-1.4***		DinG ATP-dependent helicase
dinP		***1.2***				1.6	1.7		DNA polymerase IV
hslB	2.5							2.3	HU-like DNA-binding protein

recJ	-1.6								ssDNA-specific exonuclease RecJ
recX	1.7			***1.4***					recombination regulator RecX
ssbA				3.1			1.6		single-stranded DNA-binding protein
xerD		1.5					***1.4***		integrase-recombinase
yajF		***1.4***		2.4	***1.2***		1.7		hypothetical protein
yjaF	2.4								hypothetical protein
yneB		***1.2***	***1.3***	1.8			***1.3***		hypothetical protein
dapA	1.6								dihydrodipicolinate synthase
dltB	2.8	-1.7		2.1		-1.6	-1.7	-2.4	peptidoglycan biosynthesis protein
dltD	4.1	-1.6		3.6			***-1.3***		peptidoglycan biosynthesis protein
glmS	1.8				***1.4***				glucosamine--fructose-6-phosphate aminotransferase
mscL	1.8								large-conductance mechanosensitive channel
murB	1.5		1.9						UDP-N-acetylenolpyruvoylglucosamine reductase
murQ		-1.8	***-1.5***				-1.6		N-acetylmuramic acid 6-phosphate etherase
plpA	-2.8	-2.2	-1.6	-3.0	-1.8	-1.6	-2.5		outer membrane lipoprotein
plpB		***-1.4***	***-1.2***	-2.1	***-1.5***		-1.7		outer membrane lipoprotein
plpC	-2.2	-1.6	***-1.4***	-2.4	***-1.3***		-1.8		outer membrane lipoprotein
ptk	2.7	***1.5***		1.9					phosphoketolase
tagL		2.0	***1.2***				***1.3***		exopolysaccharide biosynthesis protein
ycbH	-1.6								sugar transferase
ycbI	-1.7								sugar transferase
ycbK	-1.7								polysaccharide biosynthesis export protein
yfbI			-7.6						hypothetical protein
yfbJ			-7.2						hypothetical protein
yijB			1.7						hypothetical protein
yijC			1.7						ABC transporter permease transport and binding protein
yijH	1.8								hypothetical protein
ymjE	1.5								glycosyl transferase family protein
yncA			***1.3***	1.7					acetyltransferase

ynfC	1.5		***1.5***	***1.5***					hypothetical protein
ytbA		1.6	***1.3***		***1.2***		2.1		hypothetical protein
ytjA	1.8			***1.2***					hypothetical protein
clpE	2.4		***1.2***	***1.5***					ATP-dependent protease
gpo	1.8								glutathione peroxidase thioredoxin, glutaredoxin, and glutathione
groES	2.8								co-chaperonin GroES 10 kDa chaperonin
osmC	2.5			***1.5***					osmotically inducible protein
pmsX	2.0								peptide methionine sulfoxide reductase t
sugE		1.8	2.3	2.2	***1.3***			***1.3***	SugE protein chaperones
ybjA	1.9			***1.4***					methionine sulfoxide reductase B
yfcF		2.0	1.5	2.0	***1.3***		1.9		hypothetical protein
ynhC	1.5								hypothetical protein
ynhD			1.7						hypothetical protein
amtB						1.9	-1.7	-2.2	ammonium transporter
busAB					1.8				betaine ABC transporter permease and substrate binding protein
cadA	2.3			3.7			2.4		cadmium efflux ATPase transort and binding protein
cbiO				2.6			3.2		cobalt transporter ATP-binding subunit
feoA								1.5	ferrous ion transport protein A
mtsA				-3.2			-3.7	-3.6	manganese ABC importer
mtsB				-2.0			-2.7		manganese ABC importer
mtsC				-2.0			-2.6	-3.8	manganese ABC importer permease
pacL				2.3			2.4		magnesium-transporting ATPase
pstF	1.5								phosphate ABC transporter substrate-binding protein
yafB		2.1	1.6	2.7			2.6		sulfate permease, MFS superfamily
ybcC		1.8		1.7			1.7		MATE family efflux transporter
yceA		1.6					1.6		hypothetical protein
ydiF		***1.3***			1.7		***1.2***		Na+/H+ antiporter transort and binding protein, cations
ygfE			1.8	-5.7		-1.9	-2.7	2.0	magnesium importer and exporter
yjdJ		1.7	***1.5***	2.1			1.8		potassium channel protein transort and binding protein, cations

ymgF		1.9			1.6				hypothetical protein
ynjC		***1.4***					1.6		hypothetical protein
ynjE		1.9	1.5				1.6		hypothetical protein
ynjF					***1.3***		1.6		hypothetical protein
ypbB		1.8	***1.3***		***1.3***		2.1		magnesium transporter transport and binding protein
ytgB	2.3			2.0					cesium ABC transporter substrate binding protein
yuhE	2.5			1.5				1.8	copper homeostasis protein
yvfA		1.5					***1.5***		metal ABC transporter substrate binding protein
aldC			2.0						alpha-acetolactate decarboxylase fermentation
dltA	3.0			2.1				***-1.3***	D-alanine--poly(phosphoribitol) ligase subunit 1
dltC		-1.9		2.2			-1.8	-2.2	D-alanine--poly(phosphoribitol) ligase subunit 2
fabG2		2.1	1.9	2.1	1.6		2.6		3-oxoacyl-ACP reductase
yoaI				1.6					hypothetical protein
yrbC		***1.3***					1.8		1,4-dihydroxy-2-naphthoate octaprenyltransferase
yrbE		***1.4***	1.6				2.0		hypothetical protein
comEC		1.9	1.9				2.4		hypothetical protein competence protein
yhhD		2.0	1.7		1.5		2.6		hypothetical protein
ysbA	***-1.2***	1.6					1.6		hypothetical protein
ysbD	-2.2			***-1.3***	***-1.3***	-1.8		***-1.5***	hypothetical protein
ythC			2.4						hypothetical protein
L200065		***1.2***					2.3	1.7	hypothetical protein
yafJ		1.6	***1.3***				2.0		hypothetical protein
yaiB		1.7		1.7			2.0		hypothetical protein
yaiH			1.7		1.6	1.7			hypothetical protein
yaiI		2.4	2.0		***1.2***		2.4		hypothetical protein
yaiJ		2.6	2.2		1.8		3.2		hypothetical protein
ybeH				2.3			1.9		hypothetical protein
ybfC		***1.4***	2.0						hypothetical protein
ybgA		1.6	1.8						hypothetical protein

ycdA			1.6						hypothetical protein
yciF		1.5							hypothetical protein
yciG		1.8	***1.4***	2.1			2.3		hypothetical protein
ydbD							1.5		hypothetical protein
yeiD	2.2	***-1.3***			-2.3				hypothetical protein
yfbK		***-1.5***	-3.5		-2.1				hypothetical protein
yfiH	-1.8								hypothetical protein
yfiI	-2.0			***-1.3***					hypothetical protein
ygaD	-1.8								hypothetical protein
ygeB			***-1.2***	-1.6			-2.1		hypothetical protein
ygeC	-2.1			***-1.3***				***-1.2***	hypothetical protein
yghB						1.5			hypothetical protein
yhhA		1.8	***1.4***				2.3		hypothetical protein
yhhB							1.7		hypothetical protein
yhhC		***1.3***		2.6			***1.5***		hypothetical protein
yhjA	4.3		2.1	1.9				2.5	hypothetical protein
yhjE							-1.7		hypothetical protein
yiaC	-1.8								hypothetical protein
yiiD				2.1					hypothetical protein
yjdI		2.0	***1.5***				1.8		hypothetical protein
yjfJ	1.6								hypothetical protein
ykbC		1.8	***1.4***				2.2		hypothetical protein
ykbD		1.9	1.5				1.9		hypothetical protein
ykbE				1.6					hypothetical protein
ykcE	2.0								hypothetical protein
ykjI		-2.0					-2.3		hypothetical protein
ylhB			2.5			***1.2***			hypothetical protein
yliD		2.5	1.9		***1.4***		2.6		hypothetical protein
ymbC			1.7	2.0		1.9	1.9		hypothetical protein

ymbD		2.3	2.0	2.1			2.6		hypothetical protein
ymbH		1.7	***1.3***				1.8		hypothetical protein, putative protease
ymcA		1.6	***1.2***				2.1		hypothetical protein
ymcB		2.0	***1.5***		***1.3***		2.0		hypothetical protein
ymcC		2.2					1.6		hypothetical protein
ymeA		2.6	2.5		1.8		3.2		hypothetical protein
ymhG		***1.3***					1.8		hypothetical protein
yndE		1.7					***1.4***		hypothetical protein
yneC	4.0		2.4	3.1			***1.3***		hypothetical protein
yneG			2.6						hypothetical protein
ynfH		2.4		2.7	2.9	1.9	2.1		hypothetical protein
ynhA					***-1.3***		-2.8		hypothetical protein
yniC		***-1.3***			-1.6				hypothetical protein
yniJ		1.7	***1.3***	1.7			2.1		hypothetical protein
yofM		-2.0					-1.8		DNA-binding protein
yohD	1.7								hypothetical protein
ypaG				1.6					hypothetical protein
ypiH							1.7		hypothetical protein
ypiJ		***1.2***					1.8		hypothetical protein
ypiL		***1.2***	***1.3***	1.5			***1.3***		hypothetical protein
yqbI		***1.5***	1.6				2.0		hypothetical protein
yraE				***1.3***		1.6			hypothetical protein
yrbB					-1.7				hypothetical protein
yrbH		1.8	1.6				2.6		hypothetical protein
yreB	-1.6							***-1.2***	hypothetical protein
yreD		3.7					3.9	3.8	hypothetical protein
yseD		2.0	***1.4***	2.0			2.3		hypothetical protein
ysjF		1.8	***1.5***	2.0			2.6		hypothetical protein
ytbD	3.3		***1.4***	***1.4***					hypothetical protein

ytcD	2.3			***1.4***				***1.5***	hypothetical protein
yuaE				***-1.3***	***-1.3***	-2.1	-1.9		hypothetical protein
yueC	1.8	2.0	1.8				2.0		hypothetical protein
yuhC	1.5		***1.3***						hypothetical protein
yujF		2.4	1.9		***1.3***		2.3		hypothetical protein
yviJ		2.0	1.8	2.9					hypothetical protein
yxcD	***1.3***		1.9	***1.4***				1.5	hypothetical protein
floL	1.6		***1.3***						flotillin-like protein
kinA		***1.4***		-1.7					sensor protein kinase
kinB	1.7		***1.4***	***1.2***			***1.3***		sensor protein kinase
llrA	1.7								two-component system regulator
ybdC	-2.1			-1.5					hypothetical protein
yfgF		***1.5***	***1.3***				2.0		ABC transporter permease
yjjE	2.6								hypothetical protein
ythA	1.9	***1.2***	3.3				***1.3***		hypothetical protein
ythB			2.8				***1.2***		hypothetical protein
yudH		-1.7							hypothetical protein dihydrofolate reductase
comC		***1.4***	***1.2***				2.0		type 4 prepilin-like protein specific leader peptidase transformation
comGA		2.6	2.6		***1.4***		2.8		protein ComGA competence protein
comGB		2.5	1.8				2.6		protein ComGB competence protein
comGC		2.2		2.6			2.6		protein ComGC competence protein
comGD		2.7	1.7		1.5	1.9	1.6		protein ComGD competence protein
ftsY	1.7		1.7						hypothetical protein
lmrP	-1.9			2.5	1.5		1.8		multidrug efflux MFS transporter
ybdI					1.6	1.7	***1.4***		hypothetical protein
ybfD		1.6		2.4			1.9		transporter transport and binding protein
yfjF		***1.4***	***1.5***	2.7			1.9		membrane-bound transport protein transport and binding protein
ygfA				-2.8					ABC transporter ATP-binding protein
ygfB				-4.8					ABC transporter permease

yhcA			1.5				***1.2***		ABC transporter ATP-binding protein/permease
yhcC			***1.2***	2.0			***1.4***		hypothetical protein
yhcE		-2.1			***-1.4***		-1.7		hypothetical protein
ykjJ		***1.4***	1.5				1.8		hypothetical protein
ymdC		***1.4***					1.8		kanamycin kinase
ypbC		2.2	1.9		***1.2***		2.8		MATE family efflux transporter
ypfE		1.8	1.9				2.0		transporter transport and binding protein
ypgD		-1.7							multidrug resistance ABC transporter ATP-binding protein
yvhA	1.7		***1.2***	1.6			***1.5***		MATE family efflux transporter
yweA		1.7			***1.3***	***1.5***	2.0		membrane protein
yweE	2.1			1.5					hypothetical protein
yweF	1.8								hypothetical protein
ywiG		2.5	1.8		***1.3***		2.8		ABC-type multidrug transport system, ATPase component
yxbD		***-1.5***		3.9	3.1			-3.0	multidrug transporter transport and binding protein
yxbE		-2.2			2.2			-2.1	hypothetical protein
yxeA		1.6			1.9				hypothetical protein
choS							-1.7		choline ABC transporter permease and substrate binding protein
L0466		1.6					1.8		IS1077A transposase
L200000		1.9	1.6				2.4		hypothetical protein
L200002			-1.5	***-1.5***	***-1.2***	***-1.2***			bacteriophage bIL310 repressor
L200015			-1.9		***-1.3***	***-1.4***	***-1.3***	-1.8	hypothetical protein
L200016			***-1.3***					-1.6	hypothetical protein
L200018		-1.6	-2.2	-1.7	***-1.2***	-2.2	-2.2	-1.6	hypothetical protein
L200019			-1.8				***-1.3***		hypothetical protein
L200030		2.5	2.2		1.7	1.6	2.6		hypothetical protein
L200034		***-1.2***	-2.4	-1.6		-2.0		-1.5	hypothetical protein
L200038			-1.5		***-1.4***			***-1.5***	hypothetical protein
L200040			-2.2		***-1.4***	***-1.4***		-1.6	hypothetical protein
L200041		1.6							hypothetical protein

L200055	-6.9	-1.7	-2.2		-2.2	-2.4		hypothetical protein
L200056	-2.4	-2.0	-1.8	***-1.3***	-2.2	-2.2	***-1.3***	hypothetical protein
L200058	-3.3	***-1.5***	-2.0	***-1.5***		-2.5		hypothetical protein
L200071	1.6	***1.3***						hypothetical protein
L200072	1.9	1.9				1.8		hypothetical protein
L200073	2.4	2.2		1.8		2.7		hypothetical protein
L200074	3.6	3.3		1.9		4.5		hypothetical protein
L200075						2.3		hypothetical protein
L200077	-3.4	-3.7		-3.1	-2.8	-2.7	-2.7	hypothetical protein
L200079			1.7					hypothetical protein
tra1077B	1.7					1.8		IS1077B transposase
tra904A	2.4	***1.4***				2.9		transposase of IS904A
tra904B	2.2	***1.3***				2.7	1.9	transposase of IS904B
tra904D	2.2	***1.4***				2.7		transposase of IS904D
tra904E	***1.3***				***1.2***		1.9	transposase of IS904E
tra981A			2.0		***1.5***	1.5		transposase of IS981A
tra981E			2.0		***1.4***	***1.3***		transposase of IS981E
tra981G			1.9		***1.3***	***1.2***		transposase of IS981G
yafI	***1.5***				1.6	1.8	2.1	hypothetical protein transposon related function
yajA	1.7	1.9				1.7		transposase
yajE			1.5				***1.3***	transposase
yajG			2.2		***1.3***	***1.2***	***1.4***	transposase
ybdL	1.6							hypothetical protein transposon related function
ydhD	***1.4***	2.8			1.6	1.8	2.1	hypothetical protein transposon related function
ygcE	1.6							hypothetical protein transposon related function
yidF	1.5				1.7	1.8	2.3	hypothetical protein transposon related function
ymbI			1.5				***1.4***	transposase
ymhB			1.5				***1.4***	transposase

Table S3. Primers used in this study. Primers were designed on the basis of the *L. lactis* IL594 plasmid nucleotide sequences (NCBI accession no. HM021326, HM021327, HM021328, HM021329, HM021330, HM021331, and HM197723; http://www.ncbi.nlm.nih.gov)

Strain or primer pair	Genotypic characteristics
p1orf3zF / p1orf3zR	CTTGCTGACGAATTAGGAGTTAG/TCAGCGGTTAATAGCTTCATAC
p1orf6zF / p1orf6zR	CCTTGTTCGTTGGATTGATTAG/AGAACTCGCTTCTAAATTTGTG
p2citRzF / p2citRzR	AGGAGACAACAACATGAAAGTC/GAGCGTTTGTCTCCAATCTG
p2orf2zF / p2orf2zR	AGCTAAACAGACCGTTCAATAC/GTTTAGGACGATCGAACCAC
p3orf2tF / p3orf2tR	CAACAACAGAACGAAACACAAC/AAGTTCTGTTCTAAGAATACTGACG
p3orf3zF / p3orf3zR	TCTCAGGTGTTTATCTGGTATTTC/GACAACAAGTCCTACTCCAAAG
p4or24zF / p4or24zR	GTTTGATCTTACCTTGCTGTTG/GTTACTGGGAACCGATTACG
p4or49zF / p4or49zR	CCTTACCAATGGTGATGTAATCG/GTCAAAGATACTGGCGGTTTAG
p5cadAzF / p5cadAzR	GCATCGACGGTTGGTATTG/GTTTGGCGGCTAAGTCTAAC
p5cadCzF / p5cadCzR	GTTGATAGTCACAAGGATGGTAAG/AATTTACGCCAAGCTCCATAAG
p6orf6zF / p6orf6zR	TCAGGGAATTCTTTATTAGGTTCTG/CTTGTTCATCTACCTTAGTCAAGTATC
p6orf7zF / p6orf7zR	GATATACTGCGACTTCCAGTTG/ATTCTAATGCCACATGGTAAGG
p7or11zF / p7or11zR	ATCGAACCGCCAATCAAG/CCGTATTGATCCCAGAAGTAAG
p7orfXzF /p 7orfXzR	TGCAGATGAGTTGAGTGTTAC/TGCTACCACTTTCTACTTTACC
